# Malignant clonal evolution drives multiple myeloma cellular ecological diversity and microenvironment reprogramming

**DOI:** 10.1186/s12943-022-01648-z

**Published:** 2022-09-22

**Authors:** Yuanzheng Liang, Haiyan He, Weida Wang, Henan Wang, Shaowen Mo, Ruiying Fu, Xindi Liu, Qiong Song, Zhongjun Xia, Liang Wang

**Affiliations:** 1 Department of Hematology, Beijing Tongren Hospital, Capital Medical University, Beijing, 100730 China; 2 Department of Hematology, Myeloma & Lymphoma Center, Shanghai Changzheng Hospital, Naval Medical University, Shanghai, 200003 China; 3 State Key Laboratory of Oncology in South China, Collaborative Innovation Center for Cancer Medicine, Sun Yat-sen University Cancer Center, Guangzhou, 510060 Guangdong China; 4 Clinical Research Center, The Second Affiliated Hospital of Guangxi Medical University, Nanning, 530007 Guangxi China; 5 Intensive Care Unit, The Second Affiliated Hospital of Guangxi Medical University, Nanning, 530007 Guangxi China; 6 Department of Basic Science, YuanDong International Academy of Life Sciences, Hong Kong, 999077 China; 7 Experimental Center of BIOQGene, YuanDong International Academy of Life Sciences, Hong Kong, 999077 China

**Keywords:** Multiple myeloma, Malignant clonal evolution, Cellular ecological diversity, Microenvironment reprogramming, Cell heterogeneity

## Abstract

**Background:**

Multiple myeloma (MM) is a heterogeneous disease with different patterns of clonal evolution and a complex tumor microenvironment, representing a challenge for clinicians and pathologists to understand and dissect the contribution and impact of polyclonality on tumor progression.

**Methods:**

In this study, we established a global cell ecological landscape of the bone marrow (BM) from MM patients, combining single-cell RNA sequencing and single-molecule long-read genome sequencing data.

**Results:**

The malignant mutation event was localized to the tumor cell clusters with shared mutation of ANK1 and IFITM2 in all malignant subpopulations of all MM patients. Therefore, these two variants occur in the early stage of malignant clonal origin to mediate the malignant transformation of proplasmacytes or plasmacytes to MM cells. Tumor cell stemness index score and pseudo-sequential clonal evolution analysis can be used to divide the evolution model of MM into two clonal origins: types I and IX. Notably, clonal evolution and the tumor microenvironment showed an interactive relationship, in which the evolution process is not only selected by but also reacts to the microenvironment; thus, vesicle secretion enriches immune cells with malignant-labeled mRNA for depletion. Interestingly, microenvironmental modification exhibited significant heterogeneity among patients.

**Conclusions:**

This characterization of the malignant clonal evolution pattern of MM at the single-cell level provides a theoretical basis and scientific evidence for a personalized precision therapy strategy and further development of a potential new adjuvant strategy combining epigenetic agent and immune checkpoint blockade.

**Supplementary Information:**

The online version contains supplementary material available at 10.1186/s12943-022-01648-z.

## Background

Multiple myeloma (MM) is the most common type of plasma cell malignancy (93%), characterized by the abnormal proliferation of terminally differentiated clonal plasma cells in the bone marrow (BM), and accompanied by chromosomal instability and cytogenetic abnormalities [1–3]. The progress of MM is a multi-level and multi-stage dynamic process involving a wide range of clonal genetic variation and molecular evolutionary dynamics, which drive the heterogeneity of the tumor cell ecological composition and spatial clonal structure, mediating treatment resistance and recurrence [4–6]. Efforts to link the progress and prognosis of MM with gene resistance cloning have motivated further study into identifying the drivers of genetic variation in functionally heterogeneous cloning [7]. Nonetheless, existing studies on the clonal evolution of MM are mainly based on bulk-sequencing or computer simulation data at the tissue level of a high-volume BM aspirate [8–11]. To date, based on single-cell sequencing, Amit et al. identified resistance pathways and therapeutic targets in relapsed MM [12], and Lohr et al. revealed metabolic reprogramming as a resistance mechanism in BRAF-mutated MM [13]. However, few studies reported the clonal evolution trajectory of MM malignant cells at the single-cell level. Moreover, such analyses ignore the intratumor cell heterogeneity that is not only an important result of tumor subclonal evolution at the cellular level but also the micro-scale basis of individual heterogeneity, mediating the diverse prognostic outcomes of patients [14–16].

Existing studies have only focused on myeloma evolution with respect to primary variation and acquired secondary variation in longitudinal samples, without exploring the interaction mechanism of variation and selection [17, 18]. Variation and selection play equally important complementary and promotive roles in establishing tumor evolutionary patterns. Additionally, there remain many challenges in transforming the observational studies of rich heterogeneous cell-state phenomenology from single-cell analysis to a mechanical understanding of cancer evolution dynamics and causal mechanism patterns [19]. For example, defining a common feature to reflect the evolutionary process of a tumor would help to obtain more recognizable tumor biological characteristics toward developing better intervention strategies.

Therefore, in this study, we combined single-molecule long-read genome sequencing with single-cell RNA sequencing (scRNA-seq) to identify genomic instability events and the single-cell ecological landscape in MM patients. Bioinformatics approaches were applied for the direct reconstruction and in-depth global description of the cloning pattern of MM malignant cells in natural and drug-driven states to determine the change and selection of the microenvironment during the evolutionary process. The main findings were then verified in large-scale clinical MM samples, offering significant robustness in the potential to obtain a general framework of tumor cell evolution, replacing the current coarse-grained, step-wise, and deterministic cell and tissue models [19].

## Materials and methods

### Sample collection

Among the seven patients, three had newly diagnosed MM (NDMM), three had relapsed and/or refractory MM (RRMM), and one had CD20-positive RRMM (CD20^+^ RRMM). Among the three RRMM patients, RRMM1 was resistant to thalidomide and melphalan; RRMM2 received bortezomib, pegylated liposomal doxorubicin, cyclophosphamide, and dexamethasone but failed to respond; and RRMM3 received lenalidomide, bortezomib, pegylated liposomal doxorubicin, cyclophosphamide, and dexamethasone but relapsed after all regimens. Additionally, a patient was previously diagnosed with primary central nervous system lymphoma without BM invasion and had been in complete remission for 1 year without any treatment at the time of BM sampling and was considered as a control.

BM aspirates were collected from seven MM patients and one control donor who agreed to a multiplex library and sequencing protocol that covered all study procedures, which were approved by the Ethics Review Committee of Beijing Tongren Hospital and Sun Yat-sen University Cancer Center. All patients provided written informed consent. The clinical data of all patients are shown in Supplementary Table 1. All sequencing was performed at Biomarker Technologies Corporation (Beijing, China).

### Single-cell transcriptome profiling

Sample preparation and cDNA library construction were performed with the 10× Genomics Single Cell 3’v3.1 kit according to the manufacturer’s instructions. Based on microfluidic technology, individual cells and reagents required for the reaction were wrapped in GEM droplets with a bead (containing a cell barcode) on the microchip, and the droplets containing the cells were collected. The cells were lysed to release mRNA, which binds to the cell barcode primer on the bead to complete the reverse transcription reaction. The GEMs were broken, and cDNA was recovered and amplified by polymerase chain reaction to construct the cDNA library. The cDNA product and library concentration were detected using a Qubit 4.0 fluorescence quantification instrument, and the insert size was detected using a Qseq400 Bioanalyzer to ensure a single peak type, no spurious peak, no junction, and no primer dimer. Finally, the sample library was sequenced using the NovaSeq 6000 instrument on the Illumina platform. After identifying the cassava base, the original image file was converted into a sequence file and stored in FASTQ format.

### Single-molecule long-read sequencing and Nanopore sequencing

The sequencing procedure was performed according to standard Oxford Nanopore Technologies protocols [20]. First, high-molecular-weight genomic DNA was extracted from the BM aspirate using HiPure Tissue & Blood DNA Kit (D3018–03, Magen), and Nanodrop, Qubit, and 0.35% agarose gel electrophoresis were used for purity, concentration, and integrity quality inspection. Next, 2 μg of high-quality nucleic acid was prepared and fragmented with a G-TUBE tube, thereby breaking the genomic DNA to an average of approximately 8-kb fragments. The genomic DNA ligation reaction kit (SQK-LSK109) was used to construct the library. NEBNext FFPE DNA Repair Mix and NEBNext Ultra II End Repair/dA-Tailing Module were used to repair the damage of nucleic acid fragments, end repair, and to add A bases to the end of the fragments, thereby purifying the magnetic beads. After adding the barcode sequences and ligating the sequencing junction using Amplification Free Barcode Expansion Kit 1–12 (EXP-NBD104, Nanopore), the beads were purified to complete the library construction. Finally, the library was quantified and sequenced based on the Qubit fluorometer. For the original electrical signal obtained by sequencing, Guppy software [21] was used for neural network base calling (https://github.com/rrwick/Basecalling-comparison) to obtain the original sequence file, which was stored in FASTQ format.

### Flow cytometry

Cell-surface labeling was performed using flow cytometry to identify and verify MM malignant plasma cells. To detect the expression of cell antigens, cell size, and intracellular particle content, a 100 μL sample of fresh BM with EDTA was collected and mixed with fluorescein-labeled monoclonal antibodies (BD and Beckman Coulter Company) to the cell-surface markers including CD45, CD38, CD56, CD138, CD20, and CD19. Then, the mixture was incubated for 15 min at room temperature and protected from light. Red blood cell lysate (2 mL) was added, and incubated for 10 min at room temperature in the dark, followed by the addition of 2 mL phosphate-buffered saline (PBS), and the sample was centrifuged at 352 g for 5 min. After removing the supernatant, 3 mL PBS was added to wash and discard the supernatant, and then 300 μL PBS was added for machine detection. Finally, the results were analyzed on the FACSCanto II flow cytometer (BD) with BD FACSDiva software.

### Sequence alignment and gene quantification

Cell Ranger 5.0.1 software was used for sequence comparison and quantification of the sequencing data (per official recommendation of 10× Genomics), and the sequence read was mapped and aligned to the reads of the reference human genome reference (Hg38) using STAR software [22]. All unique gene names of the transcripts were recorded, the cells were labeled by the barcode, and the transcripts were labeled by unique molecular identifiers (UMIs) to quantify the number of cells and genes after comparison with the reference. All reads that mapped to the same gene and had the same UMI sequence were folded and different UMIs corresponding to the same gene were quantified, which produced a digital matrix for cell gene expression quantification. For all downstream analyses, we selected cells that have at least 1000 UMIs (indicating the number of captured transcripts) mapped to at least 200 unique genes and ensured that each gene is expressed in more than three cells. We excluded cells with poor viability and quality by removing more than 10% of the cells whose gene counts reflected mitochondrial genes or ribosomal RNA.

### Construction of a single-cell atlas

The IntegrateData function in the R Seurat package [23] was used to merge single-cell data, and cell clustering analysis was performed according to default parameters (http://satijalab.org/seurat/). Principal component analysis and t-distributed stochastic neighbor embedding (t-SNE) methods were used for dimensionality reduction and visualization of the clustering results, and the results were projected to a two-dimensional image, which was defined as a single-cell atlas. The “FindAllMarkers” function in the Seurat package was performed to identify the specific genes expressed in each cell cluster, with *P* < 0.05 considered statistically significant.

### Identification of cell types

For control donor BM cells, we used the SingleR package in R [24] to annotate the cell types. SingleR assigns cell identities to single-cell transcriptomes by comparison with the reference datasets of pure cell types for microarray or RNA-seq sequencing. Here, we used the previously defined single-cell transcriptome expression profile as a reference system [25]. We identified and extracted malignant plasma cells based on clinical and laboratory features of immunophenotypes in MM (CD38^+^CD56^+^CD138^+^CD19^−^CD20^−^). For non-classical CD20^+^ RRMM patients, a phenotype of CD38^+^CD56^+^CD138^+^CD19^−^CD20^+^ was used as the screening condition for malignant plasma cells. The extracted malignant plasma cells were verified using flow cytometry. Clusters were annotated based on the expression of known marker genes (Supplementary Table 2).

### Gene signatures

For label scoring of the cell cycle and cell characteristics, we adopted non-parametric and unsupervised scoring assumptions based on the expression patterns of characteristic genes, similar to a previously described gene set scoring strategy [26]. For scoring of the cell cycle and cell characteristics, an unlabeled sample gene expression matrix was used as input, which included the scRNA-seq count expression profile and log2-standardized chip expression profile of large-scale clinical MM patients or a bulk-seq count expression profile. First, the algorithm performed a non-parametric nuclear density estimation test on the overall gene expression profile. Second, based on the kernel density estimation results, the samples were sorted according to their expression levels. Next, the samples were ranked for expression levels based on the results of the nuclear density estimation, and the cell cycle and the rank statistic for each cell characteristic were calculated, similar to the Kolmogorov–Smirnov test. Finally, the cell cycle and enrichment scores of each cell feature were obtained and the output was provided as a data matrix corresponding to each sample.

The stemness score of the single-cell atlas was obtained using the TCGAanalyze_Stemness function in the R package TCGAbiolinks [27], which evaluates the degree of carcinogenic differentiation by extracting a series of marker genes to quantify the characteristics of stem cells from the transcriptional expression and epigenetic patterns of non-transformed pluripotent stem cells and their differentiated offspring using a publicly available molecular atlas [28]. Subsequently, one-class logistic regression machine learning algorithm (OCLR) was used for multi-platform analysis of these transcriptomic, methylomic, and transcription factor (TF) binding sites to obtain two independent indices of stem cell characteristics: DNA methylation-based stemness index (mDNAsi), which reflects epigenetic characteristics, and gene expression-based stemness index (mRNAsi), which reflects gene expression patterns. We applied a stemness score on gene expression patterns (mRNAsi) to the single-cell atlas of MM malignant subclones and control donor BMs to identify the evolutionary patterns of malignant origin and intratumor molecular heterogeneity in MM.

### Pseudotime analysis

The stemness score can determine the origin of the clonal evolution of MM malignant plasma cells, and pseudotime analysis can infer the trajectory of its evolution and development. We used the R package Monocle 3 [29] to reconstruct the developmental trajectory of the control donor BM single-cell atlas and simulate the evolutionary trajectory of the malignant subclone of MM. Monocle 2 was applied to simulate the developmental trajectory of the immune cells of the BM microenvironment subjected to evolutionary reprogramming of the MM malignant clone. Subsequently, Moran’s I statistic was used to identify genes expressed in complex trajectories under the malignant clonal evolution of MM. These genes may be the molecular driving force for the natural development of MM from a malignant origin and the evolution of drug resistance under drug selection. Additionally, we calculated the RNA rate (time derivative of the gene expression state) to predict the future state and final fate of a single cell, and to analyze its developmental lineage and cell dynamics using velocyto. R [30] by distinguishing between unspliced and spliced mRNAs in the single-cell atlas.

### Gene regulatory network

Single cell regulatory network inference and clustering (SCENIC) [31] was used to infer gene regulatory networks based on single-cell expression profiles and identifying cell states, providing important biological insight of the mechanism driving cell heterogeneity. To identify the internal transcriptional regulation driving force of control donor BM cell development and MM malignant clone evolution, we used the python module tool pySCENIC to analyze and reconstruct the gene regulatory network with TFs as the core.

The workflow starts by describing the input single-cell expression abundance spectrum matrix and applying a regression per-target approach (GRNBoost2) to infer the co-expression module, from which the indirect targets were pruned based on cis-regulatory motif discovery (cisTarget). Subsequently, AUcell was used to quantify the activity of these regulators by enriching and scoring the target genes of the regulators to obtain the regulon activity score (RAS). The single-cell data were further downscaled using the RAS matrix, and the regulon-specific score (RSS) was calculated based on Jensen–Shannon divergence (JS) to determine the cell cluster-specific regulon. The most specific and significant regulon was mapped to the single-cell cluster atlas and verified using massively parallel sample sequencing. Finally, the connection specificity index (CSI) matrix was calculated and the regulon was hierarchically clustered based on the CSI to define the regulon module to obtain the relationship between the regulon module and regulon and visualized based on the R package ComplexHeatmap [32].

### Intercellular communication

Three intercellular communication event identification tools were used to identify high-confidence ligand-receptor interactions between cells: CellPhoneDB [33], iTALK [34], and our newly developed tool CellCrosstalk. Based on the joint expression of multi-subunit ligand-receptor complexes to infer intercellular communication, CellPhoneDB emphasizes joint positive expression but neglects the importance of interaction in single-arm expression and marker genes. The R package iTALK compensates for this defect by prioritizing the identification of highly expressed or differentially expressed genes (DEGs) in cell clusters, which are matched through the ligand-receptor database to identify important intercellular communication events. However, when genes with high or differential expression are mapped to the ligand-receptor network, the lack of corresponding significance identification may result in a large number of false-positive events. Therefore, we developed an innovative tool for recognizing intercellular communication events named CellCrosstalk (original code: https://github.com/ydlife/CellCrosstalk), which prioritizes the identification of highly expressed genes or DEGs to ensure that independent ligands or receptors are positive in the corresponding cell clusters. Notably, the built-in ligand-receptor interaction network database of CellCrosstalk summarizes the built-in data of CellPhoneDB and iTALK. These genes were mapped to real and 1000 random walk ligand-receptor networks; the ligand-receptor interaction pair mapped between two cell clusters was recorded each time, and the relevant cells were identified based on hypergeometric tested intercellular communication events. Finally, combining the results of the three tools, the ligand-receptor interaction pair identified by any two tools was considered a high-confidence intercellular communication event.

### Biological process and pathway enrichment analysis

The R package clusterProfiler [35] was used to perform functional enrichment analysis on Gene Ontology biological processes and Kyoto Encyclopedia of Genes and Genomes pathways for related genes (*P* < 0.05).

### Genome chromosome structure variation and copy number variation (CNV)

Based on minimap2 software [36], clean sequencing data of BM aspirates from the control donor and MM patients were compared to the reference genome (Hg19) to obtain the comparison SAM file. Using open-source samtools software (https://sourceforge.net/projects/samtools/), the files were converted into BAM format and sorted. Sniffles software [37] was used to identify genomic chromosome structural variations of multiple samples, which were merged using SURVIVOR software. The CNV of whole-genome sequencing data in the BAM file was detected using the R package QDNAseq (https://github.com/ccagc/QDNAseq).

### Single-nucleotide variant (SNV) analysis

Four different GATK framework processes were used to identify SNVs (https://gatk.broadinstitute.org/hc/en-us). First, the germline short variant discovery (SNPs + Indels) process of the GATK framework was performed on clean Nanopore sequencing data from control donor BM aspirates (https://gatk.broadinstitute.org/hc/en-us/articles/360035535932-Germline-short-variant-discovery-SNPs-Indels-) to obtain a list of SNVs used as controls. Second, the sequencing data of BM aspirates from MM patients were subject to the somatic short variant discovery (SNVs + Indels) process of the GATK framework (https://gatk.broadinstitute.org/hc/en-us/articles/360035894731-Somatic-short-variant-discovery-SNVs-Indels-) to screen MM disease-associated somatic mutation information using control SNVs as a reference. Third, single-cell transcriptional profiles of control donors and MM patient BM tumor microenvironment (TME) cells were subject to the short variant discovery (SNPs + Indels) process (https://gatk.broadinstitute.org/hc/en-us/articles/360035531192-RNAseq-short-variant-discovery-SNPs-Indels-) to obtain a list of SNVs of control single cells and information on germline mutations in MM patients. Fourth, single-cell transcriptional profiles of malignant plasma cells from MM patients were applied to the RNA-seq short variant discovery (SNPs + Indels) process of on somatic mutation calling. The SNVs detected in MM patients and single-cell transcriptional profiles of malignant plasma cells, but not detected in control donors were considered to be MM malignant subclone-associated SNVs.

First, STAR was applied to map the scRNA-seq FASTQ format clean data of malignant plasma cells to the reference. Second, MergeBamAlignment MarkDuplicates was performed for data cleanup, and the data were processed using SplitNCigarReads. Subsequently, base quality recalibration was completed using BaseRecalibrator, Apply Recalibration, and AnalyzeCovariates. Mutect2 was applied to all candidate variants instead of the RNA-seq short variant discovery (SNPs + Indels) process for BM TME cells of control donors and MM patients, where HaplotypeCaller was applied for all variants. Next, GetPileupSummaries and CalculateContamination were applied to calculate contamination and Learn Orientation Bias Artifacts was completed based on the LearnReadOrientation Model. Finally, FilterMutectCalls was used to filter variants, and the implementation of annotated variants was based on Funcotator.

Some SNVs only existed in some malignant subclones, indicating their involvement in the malignant clonal evolution of MM. Although similar strategies for SNV detection have been reported previously [38–40], several false-positive and false-negative results were obtained. Therefore, we adopted the latest process recommended by the GATK framework to obtain reliable results. Based on these high-confidence SNVs, we calculated the tumor mutational burden (TMB) of MM patients at the BM aspirate and malignant subclonal levels based on the number of somatic variants per megabase in the genome [41]. The high concordance of the TMB at both levels suggests high reliability and robustness of our SNV-calling strategy.

### Estimation of CNV in single cells

Single-cell CNV was estimated using the inferCNV package (inferCNV of the Trinity CTAT Project, http://github.com/broadinstitute/inferCNV), which compares the gene expression of each tumor cell with the average expression or “normal” reference cell gene expression to determine its expression intensity, and displays the relative gene expression on each chromosome in the form of a heatmap. Compared with normal cells, MM malignant subclones always have over- or under-expression of local genome fragments. The resulting inferred malignant subclonal CNV events were highly consistent with the true CNV events detected by Nanopore sequencing of BM aspirates from MM patients, which not only validated the feasibility and reliability of the inferCNV strategy but also localized the CNV in patients to specific malignant subclones.

### Analysis of clonal evolution based on malignant cell clusters

A total of 11 MM malignant clonal clusters were identified in this study. Subsequently, based on the proposed temporal analysis, we preliminarily established that these malignant clonal clusters had two potential malignant origins. The R package fishplot was applied to trace the clonal structural evolution of these malignant plasma cell clusters (https://github.com/chrisamiller/fishplot).

### Survival analysis

To predict the prognostic potential of clonal profiles, we extracted specific marker genes with log fold change (logFC) > 1, which were used as cell characteristics to obtain non-parametric and unsupervised scores in a large-scale clinical cohort of 9574 patients with 24 independent datasets to determine the relative abundance of malignant progenitor cells. The difference in relative abundance was used as a standard score for MM malignant origin dominance to classify patients into origin types. Patients were also staged according to the relative abundance of malignant origin progenitor cells: type I and IX double positive, double negative, type I-specific positive, and type IX-specific positive. The R package survminer (https://github.com/kassambara/survminer) was used to determine the prognostic significance of the relative abundance of progenitor cells of malignant origin, the advantage score of malignant origin, and the classification of malignant origin.

### Prognostic model for MM drug resistance-related markers

Drug resistance evolution-related markers were extracted in the evolutionary pattern of MM malignant clones that were significantly highly expressed in the large-scale datasets of patients. These drug resistance markers were used for univariate Cox regression analysis of overall survival (OS) in the training cohort GSE136400, and the characteristic genes significantly correlated to prognosis were obtained (*P* < 0.01). These prognostic characteristic genes were included in multivariate Cox regression analysis for OS and relapse-free survival (RFS) to establish a prognostic model, which was tested using the R package survminer, and externally validated in independent GSE9782 and MMRF-CoMMpass cohorts (https://themmrf.org/). Time-independent receiver operator characteristic (ROC) curves were then conducted to assess the prediction performance of the prognostic model.

### Self-organizing mapping (SOM) analysis

The contribution of malignant subclones to the molecular heterogeneity of BM aspirates from MM was quantified by SOM analysis [42]. First, patient-specific marker genes in malignant subclones were identified using the R package Seurat, and their expression matrices were extracted. Each malignant subclone was divided for differential expression analysis, respectively.

A matrix of Pi values was used as the standard input to the SOM, where the row represents genes and the columns represent malignant subclones, where Pi = −log10 (adjusted P (P.adj)) × logFC. Next, the R package Kohonen (https://github.com/iamciera/SOMexample) was applied for SOM analysis, resulting in marker gene–MM patient-specific association patterns and malignant subclonal contributions of patient heterogeneous molecules. The Kohonen topology-preserving map creates a multidimensional continuous representation of “perceptual space” on a neuronal grid, where input by vector x = (× 1,..., x d). Exemplars of these vectors were repeatedly presented to the two-dimensional neuronal network organization to simulate the various stimuli experienced by the senses, which in turn condensed the labeled genes into the corresponding neural units. Thus, genes with similar patient heterogeneity contributions were organized in the same SOM grid neural unit and similar neural units were clustered in close proximity.

### Heterogeneity score

According to the strategy of Ma et al. [43], the degree of intratumor heterogeneity was measured by calculating diversity scores with gene expression profiles of malignant cells within the tumor. First, the intratumoral heterogeneity of each tumor was measured based on principal components (PCs) rather than the original gene expression profile to obtain the primary information and reduce noise. The top 30 PCs were selected for subsequent calculations based on the alignment of eigenvalues and were significant. This criterion was determined after using different numbers of PCs to determine the robustness of the diversity score calculation. Subsequently, the diversity score for each patient was calculated.

### Data availability

Expression profile data analyzed in this study were obtained from the Gene Expression Omnibus database (https://www.ncbi.nlm.nih.gov/geo/) and EMBL-EBI database (http://www.ebi.ac.uk) with accession numbers GSE136400, GSE9782, and E-TABM-1138. RNA-seq parallel sequencing data were obtained from the Multiple Myeloma Research Foundation CoMMpass Study (https://research.themmrf.org/).

The raw data of scRNA-seq and single-molecule long-read genome sequencing have been deposited in the Genome Sequence Archive in National Genomics Data Center (GSA: HRA001335) and are publicly accessible at https://ngdc.cncb.ac.cn/gsa.

## Results

### Single-cell landscape of control BM

**Fig. 1:**
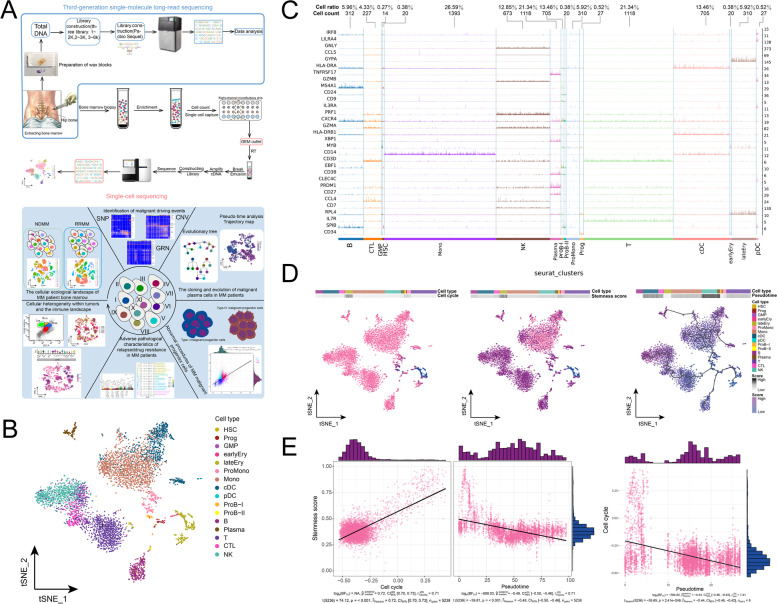
Cell population of control donor BM samples. **A** Overview of the study workflow. BM aspirates were collected and processed from control donors and MM patients for scRNA-seq and Nanopore sequencing to characterize the global single-cell ecological landscape and clonal evolution model of MM. **B** Single-cell profiles of the control donor BM based on t-SNE approach. Each color represents a cell identity, including hematopoietic lineages such as hematopoietic stem cells and juvenile red blood cell lineages, myeloid cells such as pro-monocytes and monocyte dendritic cells, and lytic cells such as T cells, B cells, and NK cells, for 16 cell types. **C** Tracks plots showing known marker genes specific to the identity of control donor BM cells. The cluster modules in the columns indicate the cell identity of the control donor BM, while the rows indicate the expression of the marker genes, along with cell abundance and cell ratio. **D** Single-cell atlas based on t-SNE showing the cell cycle score, stemness score, and pseudotime score of control donor BM cells. **E** Correlation between stemness score, cell cycle score, and pseudotime score (*p* < 0.001). MM, multiple myeloma; t-SNE, t-distributed stochastic neighbor embedding; TF, transcription factor; BM, bone marrow; GRN, gene regulation network; RSS, regulon specificity score

From scRNA-seq, 64,718 cells were obtained, including 5238 from the control donor BM aspirate and 59,480 from MM patients after quality control (Fig. 1A, Supplementary Table 3). The t-SNE approach captured 16 cell types, including hematopoietic lineages, myeloid lineage cells, and lymphoid cells, all of which belong to common cell groups in the BM (Fig. 1B), consistent with the established phenotypic characteristics of immune cells (Fig. 1C). The pseudotime trajectory of cell development revealed the continuous process of myeloid development and hematopoiesis, that hematopoietic stem cells (HSCs) differentiate into promonocytes to monocytes to finally dendritic cells (DCs), and that HSCs differentiate into erythroblasts to post erythroblasts (Fig. 1D). The pseudo-sequential differentiation trajectory was negatively correlated with the stemness and cycle scores of BM cells, which is in line with the physiological changes of cell differentiation (Fig. 1E).

The single-cell profile of the control donor was constructed to reveal the ecological composition of different hematopoietic cell types in the normal BM, suggesting differentiation trajectory and fate choice, which is consistent with the current hematopoietic concept. Thus, this concept can be applied as a training cohort and reference system for the cellular ecological landscape of MM.

### Cellular ecosystem landscape in MM

**Fig. 2:**
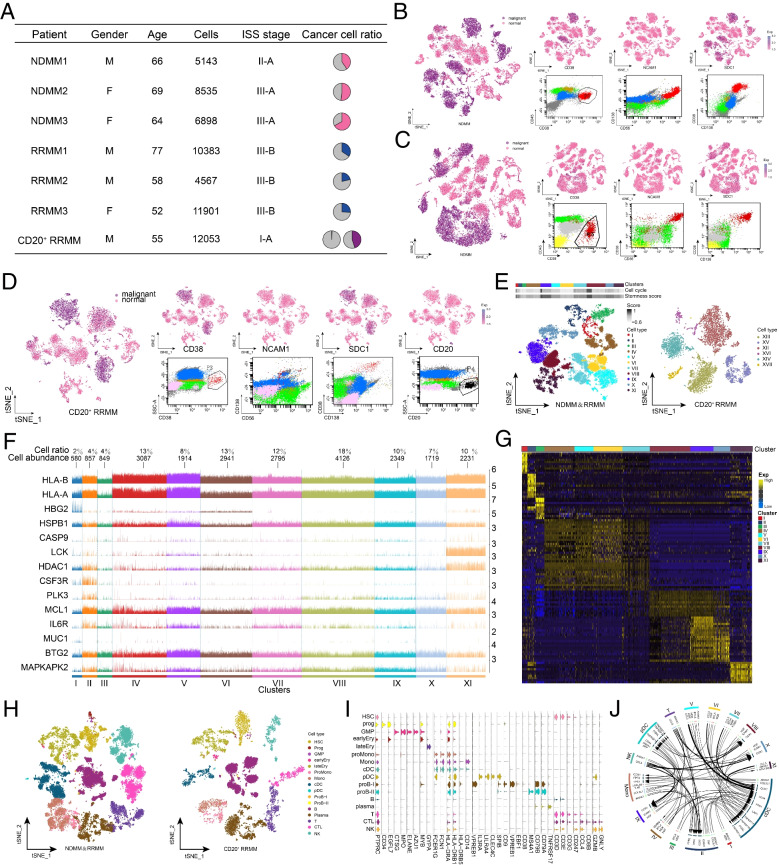
Cellular ecosystem of the BM of MM patients. **A** Clinical data and single-cell profiles of MM patients. Cell counts indicate the single-cell transcriptome that passed the quality threshold for each patient. The proportions of malignant plasma cells detected in smears, flow cytometry, and scRNA-seq in BM aspirates for each patient are demonstrated in pie charts. The two pie charts for scRNA-seq in CD20^+^ RRMM patients represent the proportion of CD20^+^ malignant plasma cells and the proportion of all malignant plasma cells, respectively. **B**-D Identification of malignant plasma cells in patients with NDMM (**B**), RRMM (**C**), and CD20^+^ RRMM (**D**). Left: Single-cell atlas of t-SNE-visualized malignant cells of the microenvironment in patients. Top right: Identification of malignant plasma cells with clinical and laboratory markers of MM malignancy such as CD38, CD56 (NCAM1), CD138 (SDC1), and CD20. Bottom right: Flow cytometry plot for validation of malignant plasma cell identification. **E** Malignant plasma cell subpopulations in patients with MM. Left: t-SNE single-cell atlas visualizing malignant plasma cell subpopulations in the BM of patients with NDMM and RRMM. The top panel shows the cell cycle score and stemness score of these malignant plasma cell subpopulations. Right: t-SNE single-cell atlas visualizing malignant plasma cell subpopulations in CD20^+^ RRMM patients. **F** Malignant marker genes shared by malignant plasma cell subpopulations in patients with NDMM and RRMM. Cluster modules in columns indicate malignant plasma cell subpopulations, while rows indicate expression of marker genes, cell abundance, and cell ratio are also shown. **G** Specific expressed marker genes for malignant plasma cell sub-clusters. **H** Microenvironment cells in the BM of patients with MM. A single-cell atlas based on t-SNE showing cells in the microenvironment in NDMM, RRMM (left), and CD20^+^ RRMM (right). **I** Expression patterns of known cell identity-specific markers in MM patient myeloid tumor microenvironment cells. Relevant marker genes were specifically expressed in the corresponding cell identity. **J** High-confidence communication network between malignant plasma cells and cells of the microenvironment in the BM of patients with MM. The Circos plot demonstrates each high-confidence ligand-receptor interaction pair of malignant plasma cell subpopulations and microenvironmental cells. The arrowheads are oriented from the ligand of the source cell toward the receptor of the target cell, while the thickness of the arrowheads represents the mean value of expression of the ligand-receptor interaction pair. NDMM, newly diagnosed multiple myeloma; RRMM, refractory or recurrent multiple myeloma; t-SNE, t-distributed stochastic neighbor embedding; BM, bone marrow

The malignant plasma cells extracted from NDMM and RRMM patients (CD38^+^CD56^+^CD138^+^CD19^−^CD20^−^; Supplementary Fig. 1A, B and Fig. 2A-C) were further clustered, with 11 malignant plasma cell clusters obtained, whereas six malignant plasma cell subsets were obtained for CD20+ RRMM patients (CD38^+^CD56^+^CD138^+^CD19^−^CD20^+^; Supplementary Fig. 1C and Fig. 2D, E). Malignant cell clusters shared cancer characteristics and widely significantly overexpressed HLA-A, HLA-B, MCL1, HDAC1, LCK, HSPB1, and IL6R (Fig. 2F), indicating a common tumor origin. Corresponding specific markers were also identified between different malignant cell clusters (Fig. 2G, Supplementary Table 4), providing direct evidence for the formation of different subpopulations.

We identified and annotated the major cell types within the microenvironment in MM patients by investigating the expression patterns of known marker genes, which enabled to accurately define the specific identity of the microenvironment cells in MM patients (Fig. 2H, I, Supplementary Table 5). There was complex and active communication between different BM cells in MM patients, regardless of newly diagnosed and relapsed patients (Supplementary Fig. 1D, E and Fig. 2J). Consequently, the global cell ecological landscape of BM from MM patients can thus be characterized and clarified.

### Variation events related to cell clonal evolution

**Fig. 3:**
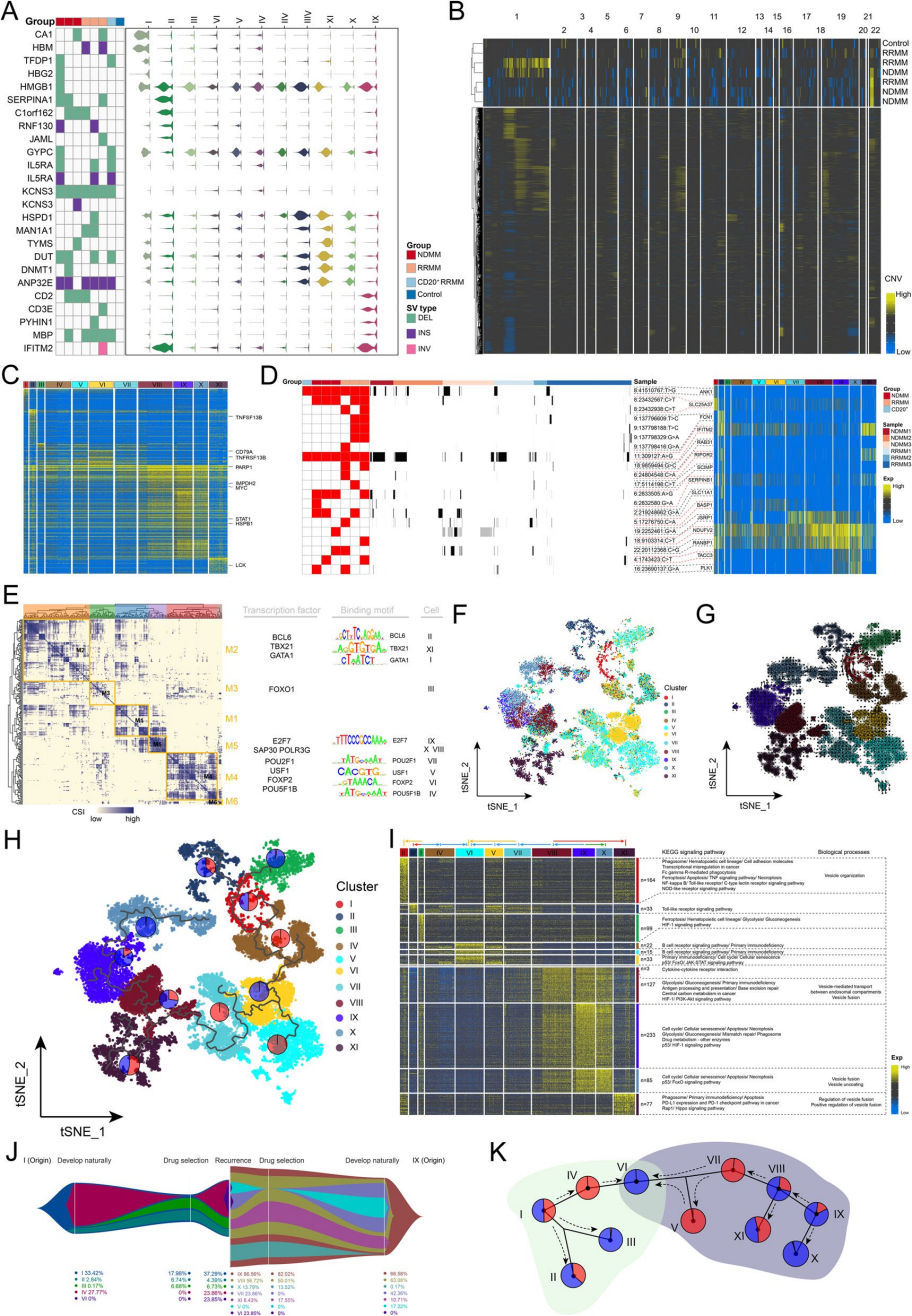
Relationship between the malignant progression of MM and the evolution of cell clones. **A** Gene transcriptional activity of SV event genes in MM malignant subclones. Left: SV spectrum of patients with MM. These SVs occur only in patients with MM, but not in control donors. Right: Expression pattern of SV genes in malignant plasma cell clusters. **B** CNV atlas of MM patients at the large-volume BM tissue level and at the single-cell level. **C** Transcriptional activity of CNV event genes in MM malignant subclones. Genes identified to be associated with the development of MM in previous studies have been highlighted. **D** Transcriptional activity of SNV event genes in MM malignant subclones. The SNV events detected simultaneously by the single-molecule long-read genome sequencing and single-cell transcriptome are demonstrated. Left: SNV mapping of patients with MM at the level of large-volume BM tissue. Middle: SNV atlas of MM patients at the level of BM monocytes. Right: Corresponding SNV gene expression patterns in the malignant plasma cell clusters. **E** Co-expression modules of transcription factors in malignant subclones of BM from patients with MM. Left: Identification of regulator modules based on the regulator’s CSI matrix. Middle: Representative transcription factors and their binding motifs in the module. Right: association of modules with malignant subclones. **F** t-SNE single-cell atlas mapping of MM malignant subclone-specific GRN. **G** RNA rate flow of MM malignant subclones mapped in t-SNE single-cell profiles. **H** Proposed chronological clonal evolutionary trajectory of MM malignant plasma cells mapped on the t-SNE single-cell atlas. The proportion of NDMM and RRMM cells characterizing the drug sensitivity of malignant cell subclones demonstrated using pie charts. The clonal evolution landscape characterizes the core state of MM malignant subclones in the malignant process with phenotypic transition differentiation. **I** Expression patterns of genes associated with the proposed chronological clonal evolution of MM malignant plasma cells and their biological signaling and cascade activation. **J** Structural changes in MM malignant plasma cells. Fish plots demonstrate the structural changes in MM malignant plasma cells from their origin through natural development, drug selection, and eventual relapse. **K** Global clonal evolutionary patterns of MM malignant plasma cells. Pie chart showing the cell proportion of NDMM and RRMM cells characterizing the drug sensitivity of malignant cell subclones. SV, structural variation; CNV, copy number variations; MM, multiple myeloma; BM, bone marrow; SNV, single nucleotide variation; CSI, connection specificity index; t-SNE, t-distributed stochastic neighbor embedding; GRN, gene regulation network

Chromosomal instability is a hallmark of human cancer and tumor heterogeneity. The structural variations catalog (Supplementary Fig. 2A) in the present study showed significant genomic instability in the majority of chromosomes, except for chr4, including deletion (DEL), duplication, inversion, and insertion (INS). Genes related to DEL and INS events were expressed and played a major role in driving malignant cell clustering (Fig. 3A). Furthermore, malignant CNV events (Fig. 3B) and the corresponding transcripts (Fig. 3C), such as TNFSF13B, CD79A, TNFRSF13B, PARP1, IMPDH2, and MYC, were identified, expressed either alone or in combination in different malignant cell subpopulations and contributing to polyclonality. IFITM2 has been proven as an effector gene of the type I interferon response that protects cells against invading viral pathogens [44]; the shared mutation of IFITM2 (chr11:309127:A > G) in all malignant subpopulations was detected at both the bulk and the single-cell levels, with high expression activity in most malignant cells (Fig. 3D), suggesting its potential as a candidate mutation of malignant origin. Similarly, the shared mutation of ANK1 (chr8:41510767:T > G) was detected in all malignant subpopulations, but only expressed in type I malignant subpopulation. These mutations were concentrated in the patterns of C > T and T > C (Supplementary Fig. 2B), which is consistent with the basic genetic concept that C in CpG dinucleotides tends to mutate to T after methylation [45, 46]. Further analysis showed that other patients had higher TMB levels than that of the CD20^+^ RRMM patient and had enriched type IX malignant cell clusters (Supplementary Fig. 2C). TMB was consistent at the bulk and the single-cell levels (Supplementary Fig. 2D). In addition to genomic variation, the gene regulatory network with TFs as pivots was organized into six modules (Fig. 3E), such as BCL6, FOXO1, E2F7, and FOXP2, to regulate the specific gene expression (Fig. 3F) and RNA transcription rate (Fig. 3G) of MM malignant cell subpopulations to guide cellular fate choice. This promotes the transformation and differentiation of the core state (Supplementary Fig. 3A), ultimately mediating the formation of a series of clonal phenotypes [30].

The pseudo-sequential clonal evolution atlas of MM malignant cells was finally constructed (Fig. 3H**)**, which is consistent with the trend of the cell stemness index and cell cycle score (Supplementary Fig. 3B, C), whose corresponding gene expression, signaling pathways, and biological functions are shown in Fig. 3I. According to the cell stemness index score and pseudo-sequential clonal evolution analysis, the malignant origins of the cells were divided into type I and type IX malignant progenitor cell origins with the highest level of cell stemness index score (Fig. 3J). During the natural development of MM cells (Fig. 3K), type I and type IX malignant progenitor cells evolved into type II, III, and IV and type VIII, X, VII, and V malignant cells, respectively, with different drug sensitivity profiles; thus, type IV, VIII, VII, and V subpopulations explosively grew to an occupied advantage. However, with the selection of drugs and the occurrence of chemical carcinogenic variants, new cloned type VI malignant plasma cells were formed and became a superior subpopulation.

### Multi-omics abnormal program identification of primitive MM malignant progenitor cells

**Fig. 4:**
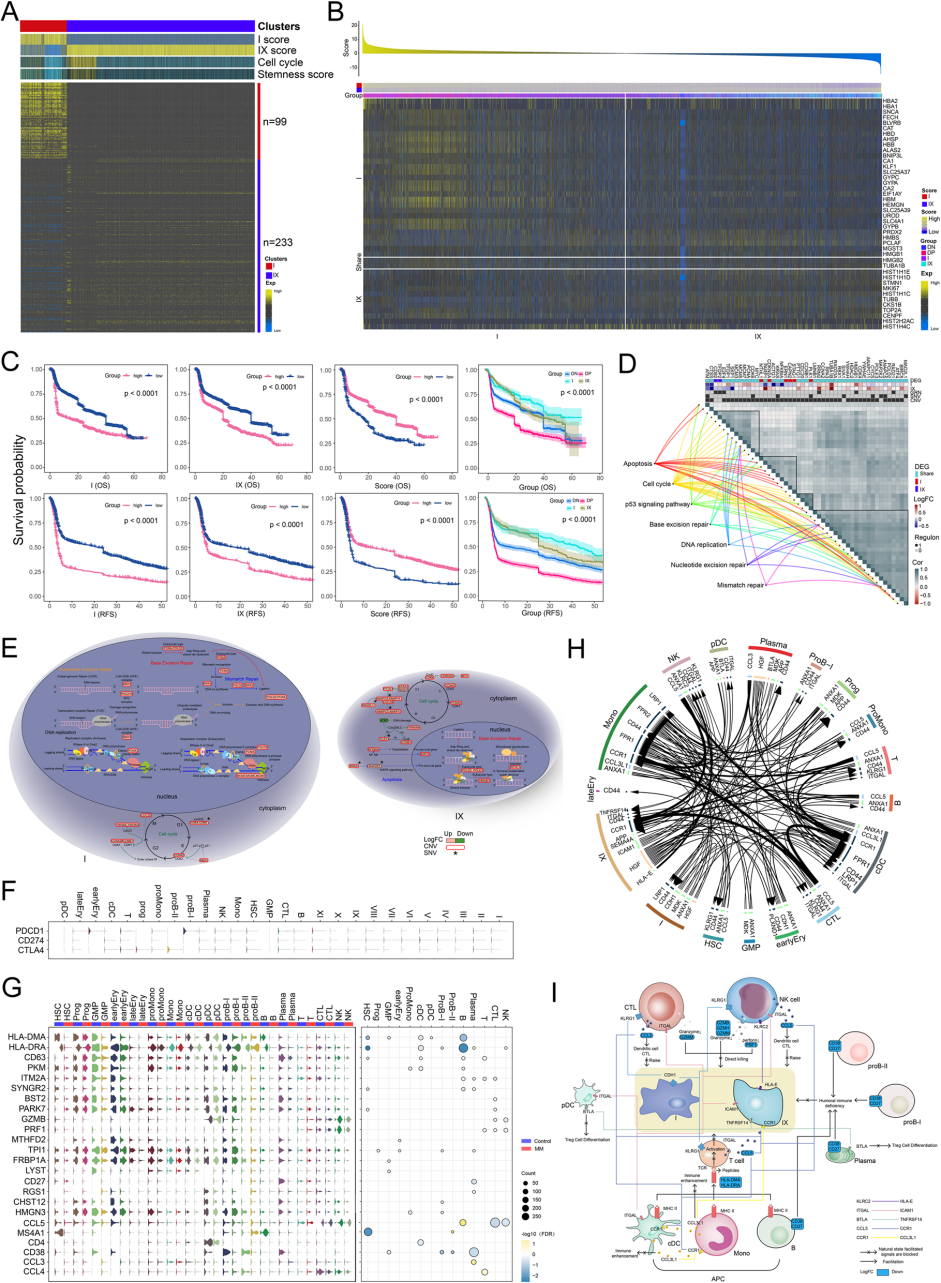
Multi-omics anomaly procedure for MM malignant origin. **A** Expression pattern characteristics of MM malignant origin. The bottom heatmap shows the expression pattern of type I and IX malignant progenitor marker genes, while the top annotations represent the GSVA score, cell cycle score, and tumor stemness score of type I and IX malignant origin. **B** Verification and typing of malignant origins in a large-scale clinical MM patient cohort. Top: malignant origin advantage score of the MM clinical patient cohort. The yellow bar represents the type I origin advantage and the blue bar represents the type IX origin advantage. Middle: abundance score of type I and type IX malignant progenitor cells in the clinical patient cohort of MM. The advantage score of malignant origin at the top = type I malignant progenitor cell abundance score - type IX malignant progenitor cell abundance score. Bottom: expression patterns of type I and type IX malignant progenitor marker genes in the MM clinical patient cohort. **C** Clinical prognostic value of MM malignant origin. Survival curves demonstrating the survival prognostic potential (OS and RFS) of type I and IX malignant progenitor abundance score, malignant origin predominance score, and malignant origin predominance typing in a cohort of patients with MM. **D** Variation in the malignant origin of MM drives a global gene expression regulatory network. The associated genes were regulated by GRN, SNV, and CNV, and clustered into four modules based on expression correlation to activate or inhibit seven biological signaling pathways. **E** Molecular mechanism of malignant origin mediated by early carcinogenic drivers. **F** Expression patterns of immune checkpoints PD-1, PD-L1, and CTLA4 in malignant plasma cells and tumor microenvironment cells in patients with MM. The negative expression of immune checkpoint-related genes in malignant plasma cells and tumor microenvironment cells provides molecular insight at the single-cell level for the poor efficacy of immune checkpoint blocker therapy. **G** Expression pattern of antitumor immune response cascade-related genes in microenvironment cells. **H** Communication network with high confidence between early malignant progenitor cells and tumor microenvironment cells in patients with MM. The Circos diagram illustrates each high-confidence ligand-receptor interaction of type I and type IX malignant progenitor cells and microenvironment cells. The direction arrow is from the ligand of the source cell to the receptor of the target cell, and the thickness of the arrow represents the average expression level of the ligand-receptor interaction. **I** Immune escape mechanism of early malignant progenitor cells in MM. MM, multiple myeloma; GRN, gene regulation network; CNV, copy number variation; SNV, single nucleotide variation; OS, overall survival; RFs, relapse-free survival

Subsequently, we focused on primitive MM cell subpopulations, including type I and IX malignant progenitor cells, which promote the occurrence and growth of tumors with high stemness and cell cycle activity (Fig. 4A). Type IX malignant progenitor cells showed higher stem cell characteristics, resulting in a higher degree of malignancy in patients. Their cell markers with logFC > 1 were extracted to verify the expression and relative abundance in a large-scale clinical cohort of 9574 patients with 24 independent datasets (Supplementary Table 6) and the MM pathological classification of malignant origin was performed (Fig. 4B, Supplementary Table 7). A higher relative abundance of malignant progenitor cells was associated with a worse prognosis (*P* < 0.0001; Fig. 4C) for both types I and IX, especially for the latter (P < 0.0001). We observed similar results at the single-cell level with high robustness. Patients with a double-positive origin of type I and IX had the worst prognosis, followed by those with a double-negative origin (P < 0.0001). Consistent with the relative abundance analysis, the prognosis of patients positive only for type IX origin was worse than that for only type I origin.

We evaluated the malignant forerunner events that were preferentially highly expressed by two malignant origin progenitor cells, including DNA damage repair, cell cycle, proliferation, migration, invasion, and stemness (Supplementary Fig. 3D). In this regard, they showed extensive similarities, with many previously reported hematological tumor-related genes, such as HMGB1, CCND2, CDK1, CDKN2A, and MYC [47–51]. These genes were more significantly dysregulated in type IX malignant cells, which explains the higher stem cell characteristics and malignant degree in the type IX origin. The similarity of malignant origin plasma cells also lies in their significantly dysregulated signaling pathways (Supplementary Fig. 3E). It is noteworthy that the type I malignant origin was significantly enriched in both base excision repair and DNA damage repair to reduce random variation events in the process of malignant proliferation, while type IX only activated the base excision repair, causing more random variation events in the process of clonal proliferation with a richer clonal pattern. Related dysregulated genes were clustered into co-expression modules according to expression similarity, which are mainly regulated by CNV events, involving SNVs and TFs (Fig. 4D), resulting in a shared carcinogenic initiation program and its own specific carcinogenic regulation mechanisms in two malignant origins of MM (Fig. 4E). Homogeneity and specificity are reflected not only in the carcinogenic processes of these origin types but also in the change in the expression pattern of their evolution (Supplementary Fig. 3F) with joint high expression of HBG2, MYC, CD79B, and MCL1 (Supplementary Fig. 3G). By contrast, the type I origin will reduce the expression of CDK6, ITGB1, BCL2L1, and STAT1 during evolution, whereas the type IX origin will promote their expression. Additionally, the type IX origin shows precursory potential for further evolution, in which the local cell population takes the lead in showing the gene expression pattern of subclone type VIII, with higher evolution efficiency. In contrast, the type I origin maintained a similar expression pattern in some cell populations cloned by the offspring, resulting in relatively slower evolution. These different evolutionary transition forms and efficiency indicate that the type IX origin can evolve more cell subclonal clusters than the type I origin within the same timeframe.

We further explored early immune escape mechanisms, as the foundation for immune resistance and evasion, throughout the malignant process of MM. Immune checkpoints PD-1, PD-L1, and CTLA4 were negatively expressed in malignant plasma and microenvironment cells (Fig. 4F). The expression pattern of genes related to tumor immune killing in the microenvironment cells (Fig. 4G) was used to build a high-confidence immune escape intercellular communication network in the early stage of MM malignant transformation (Fig. 4H), providing insight into the potential mechanism (Fig. 4I) from innate to specific immunity. Among them, HLA-DMA and HLA-DRA were generally missing in antigen-presenting cells (B cells, classical dendritic cells (cDCs), and monocytes), which is the first weakness in the immune response cascade against MM (P.adj < 0.0001). CCL5 expression was also inhibited in CTLs and NK cells (P.adj < 0.0001), which significantly reduces the efficiency of recruiting DCs and more CTLs into the nidus [52]. Additionally, defective expression of the perforin PRF1 and the granzyme GZMB in NK cells is the main reason for the inherent antitumor immune inactivation in MM patients (P.adj < 0.0001). Moreover, the expression of CD38 and CD27 in the B cell lineage (B, ProgB-I, ProgB-II, and plasma) was inhibited (P.adj < 0.0001), indicating deficiency of humoral immunity, while low levels of CCL3L1 also suggest low immune activity of monocytes (P.adj < 0.0001) [53].

### Relapse and drug resistance in MM patients at the single-cell level

**Fig. 5:**
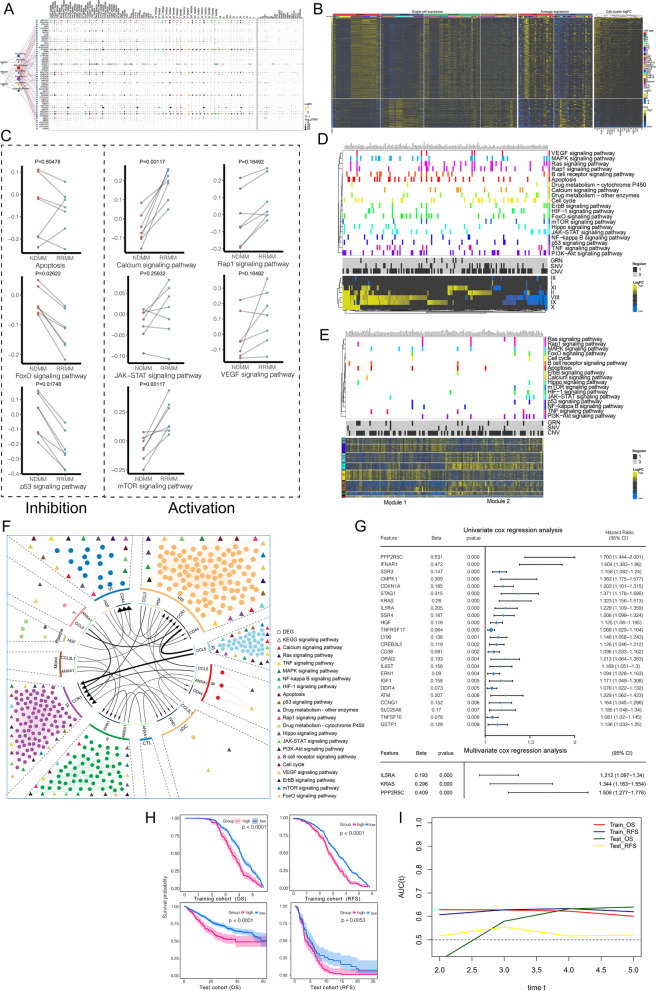
Adverse pathological features of drug resistance recurrence in MM patients observed at the single-cell level. **A** Expression patterns of drug targets in malignant subclones and tumor microenvironment cells. Left: Bubble size represents the number of drugs with resistance in patients with RRMM. The connecting lines are colored according to the drug. Middle: Bubbles of different colors represent the tolerance of RRMM patients to different drugs, and the size represents the number of targets. Right: Expression of drug targets in malignant subclones and tumor microenvironment cells. **B** Single-cell contribution of DEGs in RRMM patients compared with NDMM patients. DEGs are expressed at the single-cell level (left), the average expression pattern at the cell cluster level (middle), and the differential expression (logFC) at the cell cluster level (right) in the malignant subclones and tumor microenvironment of NDMM and RRMM patients. **C** Biological signals related to drug resistance. These biological signals were significantly activated and inhibited in RRMM patients compared with NDMM patients in the six shared malignant subclones. Inhibitory signals included apoptosis, FoxO, and p53. The activated signals involved signals related to survival, proliferation, migration, and stem cell characteristics, such as calcium/Rap1/JAK-STAT/VEGF/mTOR signaling. Each edge represents a comparison of the NDMM and RRMM groups for any of the six shared malignant subclones with correlation signal scores in types I, II, III, VIII, IX, X, and XI. **D** Drug resistance-related genes significantly involved in signaling pathways. The DEGs of shared malignant subclones in RRMM patients compared with NDMM patients are identified as drug resistance-related genes. Top: DEGs and their significant signaling pathways. Middle: malignant driver of DEGs involving GRN, SNV, and CNV. Bottom: DEGs expression changes in six shared malignant subclones. **E** Clonal evolution mediates the signaling pathways significantly involved in drug resistance-related genes. Top: MM malignant clonal evolution mediates drug resistance-related genes and their biological signals. Middle: Malignant drivers of malignant clonal evolution of MM involving GRN, SNV, and CNV. Bottom: Drug resistance-related genes clustered into two gene expression modules during the evolution of MM malignant clones. **F** Comprehensive regulatory network of drug resistance in patients with RRMM. MM malignant subclones not only inhibit their own apoptosis-related signals and activate their own survival-promoting, proliferation, migration, and stem cell characteristics related signals but also reprogram microenvironment immune cells through intercellular communication, driving the latter to activate drug metabolism signals, thereby improving the drug microenvironment and survival probability. **G** Prognostic potential of drug resistance-related genes in a large-scale MM clinical patient training cohort. **H** Prognostic value of drug resistance-related genes in a large-scale MM clinical patient cohort based on the multivariate Cox model. **I** Time-independent ROC curves for evaluating the prediction performance of the prognostic model in MM patients’ OS and RFS. NDMM, newly diagnosed multiple myeloma; RRMM, refractory or recurrent multiple myeloma; GRN, gene regulation network; CNV, copy number variations; SNV, single nucleotide variation; DEG, differentially expressed gene; OS, overall survival; RFS, recurrence-free survival

We evaluated the inhibitory effect of drugs on the expression of the established target genes at the patient level (Fig. 5A) to infer the part of the drug action that failed. The expression levels of target genes for thalidomide, melphalan, lenalidomide, and cyclophosphamide in the RRMM group did not show significant differences from those of the NDMM group, whereas those for dexamethasone, bortezomib, and doxorubicin decreased in sectional cell subpopulations. It is clear that the drug resistance of RRMM1 mainly occurs prior to the action on target genes, whereas the drug resistance of RRMM2 mainly occurs after interaction with the target gene. RRMM3 shares these two drug resistance mechanisms due to the extensive application of drugs. The characteristics of drug sensitivity at the patient level are also reflected at the cell subpopulation level, as demonstrated by systematic comparison between the cell subpopulations of the RRMM and NDMM groups (Fig. 1A, Fig. 5B). In addition, we observed the increased expression of genes such as IGHG3, IGLC2, and IGHG2 and the decreased expression of genes such as IGHG4, ITM2C, and IGHA1, which mediates the formation and development of malignant clones, causing heterogeneity in patients. The signal score of malignant cells was applied to estimate the pathway activity (Fig. 5C), in which apoptosis and the FOXO/p53 signaling pathways were inhibited in RRMM, while the calcium/Rap1/JAK-STAT/VEGF/mTOR signaling pathways related to survival, proliferation, migration, and stem cell characteristics were activated, which are mainly driven by CNV events supplemented by SNVs and TFs (Fig. 5D). This is similar to the pattern of the malignant origin. During clonal evolution of malignant cells to acquire drug resistance, the transformation of gene expression (Fig. 5E) was clustered into two gene expression modules, involving four evolutionary patterns. The acquisition and maintenance of the drug resistance phenotype of malignant cells require high expression of gene expression module 1 (related to drug sensitivity) and relatively low expression of gene expression module 2 (related to the development advantage in the natural state). The genes of both modules are related to the above-mentioned drug resistance signals, which are significantly regulated by CNV events as the main driving force for the clonal evolution of drug resistance.

The global regulatory network of intercellular communication (Fig. 5F) showed that activation of the drug metabolism signal pathway in B cells and cDCs may be reprogrammed by malignant cells, whereas the drug resistance evolution of malignant cells may involve microenvironment cells such as B cells, plasma cells, T cells, and cDCs. Genes in the drug resistance global regulatory network also showed significant prognostic potential in the training cohort (Fig. 5G). Among them, IL5RA, KRAS, and PPP2R5C were independently linked to prognosis (Fig. 5G); thus, a drug resistance-related prognostic model for MM based on multivariate Cox regression analysis was established for OS and RFS, which was verified in an independent cohort (Fig. 5H). Time-independent ROC curves demonstrated that the prognostic model enabled to accurately predict MM patients’ OS and RFS (Fig. 5I).

### Intratumoral cellular heterogeneity in MM patients

**Fig. 6:**
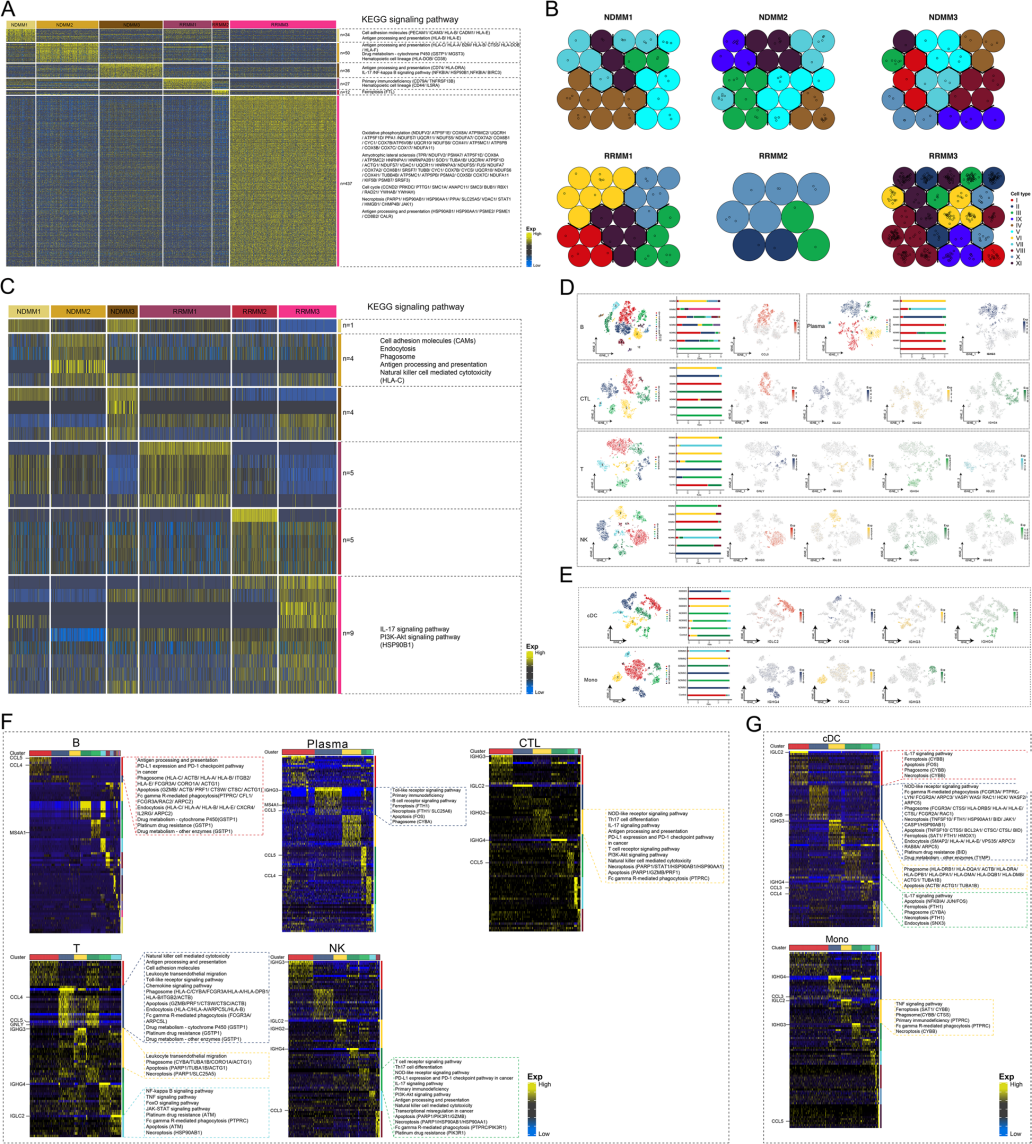
The MM malignant clonal evolution reprogramming tumor microenvironment mediates tumor heterogeneity in patients. **A** Patient-specific transcriptional patterns of MM malignant subclones. **B** Malignant subclonal self-organization contribution of specific marker genes in MM patients. **C** Patient-specific transcriptional patterns of MM BM microenvironment cells. **D** Lymphocyte subsets of the BM microenvironment of control donors and MM patients. The atlas involves B cells, plasma cells, CTLs, T cells, and NK cells. Left: Single-cell subpopulation atlas of each cell type. Middle: Proportion of each cell subgroup in the control donor and different MM patients. Right: Expression pattern of cell subgroup-specific markers mapped in the single-cell atlas. **E** Atlas of myeloid cell subsets in the BM microenvironment of control donors and MM patients. The atlas involves classical dendritic cells (cDCs) and monocyte cells. Left: Single-cell subpopulation atlas of the cell type. Middle: Proportion of each cell subgroup in the control donor and different MM patients. Right: Expression pattern of cell subgroup-specific markers mapped in the single-cell atlas. **F** Expression patterns of lymphocyte subset-specific markers and the biological signals involved. **G** Expression patterns of myeloid cell subset-specific markers and the biological signals involved. CTL, cytotoxic T lymphocytes; MM, multiple myeloma; BM, bone marrow

We applied the tumor cell-specific transcriptional diversity score to measure the intratumoral heterogeneity of MM (Supplementary Fig. 4A), detailed in the single-cell profile map of malignant cells from each patient (Supplementary Fig. 4B), which demonstrates diverse and specific ecological components with vague traces of two malignant origins. Most of the malignant cells in MM patients exhibited the activated antigen processing and presentation (Fig. 6A), although the genes involved were different, indicating that malignant plasma cells retained incomplete antigen presentation ability to a certain extent. In particular, compared with other patients, for patient RRMM3, more distinctive malignant cell ecological components were found, regulated by TFs such as E2F7, E2F8, and SAP30 (Supplementary Fig. 4C) and leading to greater heterogeneity. SOM analysis showed the contribution (Fig. 6B) and concrete manifestations (Supplementary Fig. 4D) of malignant cell subpopulations to patient heterogeneity, of which NDMM and RRMM patients showed distinct SOM neural units, suggesting that different gene clusters respond differently to the drug sensitivity of MM. Compared with malignant cells, patient heterogeneity of microenvironment cells was relatively obscure (Supplementary Fig. 4E), which was also observed at the molecular level (Fig. 6C).

### Immune cell subpopulation atlas of MM

The single-cell profiles (Fig. 6D) of B, plasma, CTLs, T, and NK cells were obtained by clustering the immune cell subpopulations, so that the heterogeneity of the immune microenvironment in MM patients was amplified at the subpopulation level. Interestingly, we found that plasma, CTLs, T, and NK cells highly expressed markers of MM malignant clonal evolution as subpopulation-specific markers, reflecting the immune cell heterogeneity of patients. Among them, IGHG3 was mainly expressed in lymphocytes of RRMM1, involving plasma-1, CTL-0, T-2, and NK-0, whereas IGLC2 was specifically expressed only in RRMM2 lymphocytes, related to CTL-1, T-5, and NK-2. Alternatively, IGHG2 was specifically expressed in the lymphocytes of CTL-2 and NK-4 in RRMM3. IGHG4 was specifically expressed in the lymphocytes of NDMM1 and NDMM3, touching upon CTL-3, T-4, and NK-4. The molecular specific markers of these microenvironment lymphocyte subpopulations are consistent with the distinctive expression of patient heterogeneous malignant single cells (Supplementary Fig. 4D). This provides strong evidence that clonal evolution of MM can reprogram lymphocyte-mediated patient heterogeneity, inspiring the novel strategy of individualized treatment in clinics. As this is an unprecedented discovery, we ruled out the possibility of wrong cell type identification for the sake of caution. The CTLs, T, and NK cells showed positive expression of their corresponding specific marker genes, which have significant cell co-localization with the corresponding patient heterogeneous malignant marker immunoglobulins (Supplementary Fig. 5A-C). Importantly, the same phenomenon was observed in cDCs, monocytes (Fig. 6E), and HSCs (Supplementary Fig. 5D). The transformation of these cell phenotypes from normal to depleted was accompanied with transformation of a series of gene expression patterns (Supplementary Fig. 5E, F) with various degrees of cell death signal activation, involving ferroptosis, necroptosis, and apoptosis **(**Fig. 6F, G). This raises the question of how malignant plasma cell markers appear in microenvironment cells to reprogram the microenvironment. By reviewing the biological signals activated by these depleted cells, we found that they not only activated various cell death signals, but also activated phagocytosis-related pathways, such as phagosome, Fc gamma, R-mediated phagocytosis (Fig. 6F, G). Correspondingly, during the clonal evolution of MM malignant cells, the key gene of exosome synthesis CD63 was expressed and a series of biological pathways related to vesicle synthesis and secretion were activated to varying degrees (Supplementary Fig. 5G, Fig. 3I). It is axiomatic that the MM malignant cells reprogramming microenvironment to augment immune escape is involved in vesicle synthesis and secretion of malignant cells as well as phagocytosis of microenvironment cells during the clonal evolution process, resulting in the emergence of malignant marker mRNA and mediating apoptosis and depletion.

In contrast, the positive expression of GNLY in T-1 emphasizes the potential malignant events that effectively activate the immune response of T cells. T-1 mainly existed in NDMM, concentrated in NDMM2, which may be related to the negative expression of malignant clonal evolution markers in the lymphocytes of NDMM2 patients, further indicating that chemoresistance of MM not only resists drugs but also inhibits immune killing.

Importantly, we found the negative expression of malignant clonal evolution markers in B cells, implying certain defensive means of B cells to resist the reprogramming and transformation during malignant cell clonal evolution, suggesting that a cellular immunotherapy strategy may benefit MM patients. CCL5 and CCL4 were specifically expressed in B-0 (Fig. 6D, F), which activate antigen presentation pathways and PD-L1/PD-1 immune checkpoints in cancer to recover the adverse effect of low CCL5 expression reducing the recruitment efficiency for cells in immune killing cascades (e.g., cDCs and CTLs). Surprisingly, B-0 were widely distributed in MM patients, mostly in the NDMM group (concentrated in NDMM2 patients), but not in the control, suggesting that an immunotherapy strategy of injecting CCL5-positive B cells derived from autologous expansion in vitro may be beneficial. Likewise, T-1 with potential immune killing efficacy highly expressed CCL5 and CCL4, activating antigen processing/presentation and chemokine signaling pathways to augment leukocyte transdermal migration. There are similar patterns of molecular characteristics and gene expression of T-1 in T-3 that were specific to controls, further highlighting the important role of CCL5 and CCL4 in the antitumor immunity of MM.

## Discussion

The unique challenges of complexity of tumor cell ecology have hindered progress in clonal evolution and intratumoral heterogeneity of MM. However, high-resolution single-cell sequencing technology has enabled the study of tumor evolution. In this study, we characterized the tumor cell and microenvironment cell ecosystem for the BM of MM patients, clarifying the potential of malignant clonal evolution patterns and their reprogramming microenvironment cells.

Moreover, we observed that from the beginning of malignant origin, the shared mutation of IFITM2 in all malignant subpopulations enhances its expression. Highly expressed IFITM2 participates in the type I interferon response to protect cells from viral pathogens and activates the expression and secretion of IL-6, which promotes the differentiation of B cells into plasma cells, mediating the growth of myeloma and stimulating bone resorption [44, 54]. Meanwhile, the activation of IFITM2 and IL-6 forms a reciprocally positive feedback loop to make MM malignant progenitor cells act out the expression pattern with virus infection. This builds a suitable host environment and microenvironment for the virus that is conducive to viral infection, such as human herpesvirus type 8 (HHV-8), thus increasing the susceptibility risk by approximately 10-fold [55–57]. HHV-8 and other viruses can continuously expand and encode the homolog of IL-6 in the host malignant plasma cells, which further activates the positive feedback loop to promote viral spread and the malignant process of MM [58]. Notably, the viral susceptibility caused by this positive feedback loop is a high-risk factor, leading to the serious consequences and high mortality of MM, similar to the recent outbreak of SARS-CoV-2 [59, 60]. Likewise, ANK1 was also identified to mutate in the early origin stage of MM, resulting in the presence of the mutation in all malignant subpopulations. ANK1 participates in the malignant progression of acute myeloid leukemia in the elderly and in children with Down syndrome, related to erythropoietin, which provides a potential molecular link for MM secondary acute myeloid leukemia and mediating poor prognosis [4, 59, 60]. In particular, according to the tumor cell stemness index score and pseudo-sequential clonal evolution analysis, the malignant origins were divided into type I and IX malignant progenitor cells. Compared with the type I origin, more significant stem cell characteristics were found in type IX malignant progenitor cells, leading to a more severe degree of malignancy and progression. It is becoming increasingly clear that MM is not a single clonal genome, but rather distinct subclones that evolve from one or more origins in the disease period, leading to intratumoral heterogeneity and different performance in environmental adaptability. The increase in clonality also depends on the accumulation of gene mutations in offspring cells to regulate the environmental adaptive competition between malignant cell subpopulations. A portion of malignant cells maintain the ability for self-renewal and long-term clonal maintenance, becoming the dominant population in the current state, while others are in a dormant state [61, 62].

Significantly, the clonal evolution of MM malignant plasma cells is not only under natural selection from the microenvironment, but also capable of reacting to the TME to create more suitable conditions for survival, including the reprogramming of immune cells to inhibit immune killing and promote drug metabolism through vesicle synthesis and secretion of malignant plasma cells as well as the phagocytosis of microenvironment cells, which has been reported in other tumors [63, 64]. Interestingly, the microenvironment reprogramming produced by clonal evolution showed significant patient specificity, mediating the tumor heterogeneity of patients, which is an unprecedented discovery that warrants full consideration of personalized and accurate treatment in clinical immune salvation therapy for MM. Specifically, the BM microenvironment of NDMM2 patients with abundant CCL5-positive B and T cells can be protected from reprogramming with an antagonistic response; thus, relevant drugs and cell therapy schemes may be of great benefit. According to previous studies, CCL5 should also be expressed in malignant cells to promote the secretion of CXCL9 by immune cells through epigenetic regulation, thus jointly recruiting T cells, whose infiltration and activation exert immune killing effects to inhibit tumor progression [65]; ligand-receptor interaction between CCL5 and CCR1 is notably active in the monocyte-plasma communication in MM patients with stage III [66]. However, we found negative expression of CCL5 in almost all of the malignant plasma cells of MM patients, which proved to be the main cause of insufficient CD8^+^ T cell infiltration and exhaustion, rather than the role of classical immune checkpoints, such as PD-1, PD-L1, and CTLA4. This emphasizes that the immune escape mediated by the downregulation of CCL5 expression occurs not only in solid tumors but also in MM, offering a new biomarker and candidate target of MM immunotherapy. As a result, a new adjuvant strategy to improve the sensitivity of immune checkpoint inhibitors for MM by jointly reversing CCL5 silencing drugs such as decitabine may be effective [65]. The new adjuvant strategy extends the indications of therapy combined with epigenetic agent and immune checkpoint blockage in MM for further treatment of RRMM. Decitabine combined with PD-1/PD-L1 inhibitors has recently entered clinical trials for patients with hematological tumors such as relapsed/refractory classic Hodgkin lymphoma, which achieved satisfactory benefits with high safety [67]. Alternatively, B cells have not been reprogrammed as other microenvironment immune cells with potential defensive measures, which warrants further research as a novel immunotherapy strategy.

## Conclusion

In summary, we explored the clonal evolution of MM occurrence and development, mediating intratumoral cell heterogeneity, and the connectivity from initial treatment to drug resistance. We then identified the interaction and response between malignant plasma cells and the microenvironment during clonal evolution. Furthermore, this study broadens the cognitive boundary of MM; however, due to the limited sample size, a larger prospective cohort is required to obtain more universal and versatile conclusions.

## Supplementary Information

**Additional file 1: Supplementary Fig. 1.** BM intercellular communication events in patients with NDMM and RRMM. (A and B) Expression patterns of CD19 and CD20 in single cells from NDMM (A) and RRMM (B) patients. Top: t-SNE single-cell atlas showing the expression of CD19 and CD20 in BM single cells from MM patients. Bottom: Verification of the expression of CD19 and CD20 in BM cells by flow cytometry. (C) Expression pattern of CD19 in single cells from CD20^+^ RRMM patients. Top: t-SNE single-cell atlas showing the expression of CD19 in BM single cells from CD20^+^ RRMM patients. Bottom: Verification of CD19 expression in BM cells from CD20^+^ RRMM patients by flow cytometry. (D) High-confidence receptor-ligand interaction for BM intercellular communication events in patients with NDMM and RRMM. Bubbles represent the average expression of high-confidence ligand-receptor interaction pairs in the source and target cells. (E) Overview of high-confidence intercellular communication networks in BM patients with NDMM and RRMM. Each bubble represents a cellular identity of the BM in NDMM and RRMM patients, and the coloring is consistent with that of the corresponding single-cell atlas. Bubble size represents active cell communication with other cells. Each arrow represents the interaction between the source cell ligand and the target cell receptor, and its thickness represents the number of ligand-receptor interaction pairs. All communication events were detected using any two tools among CellPhoneDB, iTalk, and CellCrosstalk, which represent the confidence of the reciprocal pair. BM, bone marrow; NDMM, newly diagnosed multiple myeloma; RRMM, relapsed and/or refractory MM; t-SNE, t-distribution and stochastic neighbor embedding.

**Additional file 2: Supplementary Fig. 2.** Nanopore sequencing combined with single-cell transcriptome to identify malignant driving events. (A) Diagram of genomic SVs in patients with NDMM and RRMM. “INS” and “INV” of patients with counts > 5 are displayed, and colors on chromosomes represent the number of patients with DEL (green to yellow) and DUP (green to blue). (B) Patterns of gene mutations in MM patients. (C) Volume and single-cell levels of the TMB in the BM of MM patients. Top: TMB levels of different malignant subclones. Middle: Gene mutation patterns in different malignant subclones. Bottom: TMB horizontal curve of patients with MM. (D) Correlation of TMB in MM patients at the bulk and single-cell levels. The BM TMB of MM patients was significantly correlated at the bulk level and at the single-cell level (*P* < 0.001), demonstrating the feasibility and reliability of detecting the SNV spectrum and TMB level at the single-cell level. SV, structural variation; INS, insert; INV, inversion; DEL, deletion; DUP, duplication; CNV, copy number variation; SNV, single nucleotide variation; TMB, tumor mutational burden.

**Additional file 3: Supplementary Fig. 3.** Exploring malignant origins from the perspective of clonal evolution. (A) Pseudotime trajectory of myeloid malignant subclones in NDMM and RRMM patients mapped in the t-SNE–based single-cell atlas. (B, C) Stemness score (B) and cell cycle score (C) of myeloid malignant subclones in patients with NDMM and RRMM. Top: Comparison of stemness and cell cycle scores between myeloid malignant subclones from patients with NDMM and RRMM. Bottom: Stemness and cell cycle scores of myeloid malignant subclones in NDMM and RRMM patients mapped using the t-SNE single-cell atlas. (D) Dysregulated expression patterns of malignant leader genes in type I and IX malignant origins compared to normal B cells. (E) Type I and IX malignant origin expression dysregulated genes are significantly involved in malignant precursor biological signals. These biological signals of malignant precursors can be grouped into five modules according to shared gene members. Each module was assigned an independent color. (F) Gene expression patterns of the evolution of malignant origins toward dominant subclones during natural development. (G) Expression patterns of dysregulated genes across Type I, IV, VIII and IX.

**Additional file 4: Supplementary Fig. 4.** Cellular heterogeneity in the myeloma of MM patients. (A) Heterogeneity score of the myeloma in NDMM and RRMM patients. (B) Single-cell atlas based on t-SNE showing heterogeneity of malignant subclonal ecological components in different MM patients. (C) Specific markers of malignant subclones in patients with NDMM and RRMM. Left: Expression pattern of malignant subclonal-specific marker genes in patients. Right: TFs that regulate malignant subclonal-specific markers. (D) Contribution of cell subsets with malignant subclone-specific markers in patients. Left: Expression patterns of malignant subclonal-specific marker genes in patients. The malignant subclonal marker genes illustrated are those that are the most significant and associated with subsequent MM malignant plasma cell clonal evolution reprogramming of immune cells in the microenvironment. Right: Contribution of cell subsets specifically labeled by the top specific marker malignant subclones in patients. (E) The single-cell atlas based on t-SNE showing the heterogeneity of cell ecological components in the myeloma microenvironment in different MM patients. TF, transcription factor.

**Additional file 5: Supplementary Fig. 5.** Expression patterns of immune cell subpopulations in MM. (A-C) Expression patterns of known cell identity-specific markers and their co-location with malignant markers in lymphocytes provide evidence for MM reprogramming of microenvironment cells. The pie chart shows the co-location between known cell identity-specific markers and corresponding patient heterogeneous malignant marker immunoglobulins. (D) HSC subset profiles of control donor BM and the MM patient BM microenvironment. Left: Single-cell subpopulation atlas of HSCs. Middle: Proportion of HSCs in control donors and different MM patients. Right: Expression patterns of cell subset-specific markers mapped on the HSC single-cell atlas. (E, F) MM malignant clonal evolution reprograms pseudotime trajectories and expression pattern changes in lymphoid (E) and myeloid cells (F). Lymphocytes include B cells, plasma, CTLs, T cells, and NK cells, whereas myeloid cells include cDCs and monocytes. For each cell type, the pseudotime trajectory (left), pseudotime value change (middle), and expression pattern change (right) from normal cells to MM during MM malignant clonal evolution reprogramming are shown for lymphocyte and myeloid cell subsets. (G) Expression patterns of the exosome-specific marker gene CD63 in malignant cells. HSCs, hematopoietic stem cells; CTLs, cytotoxic T lymphocytes; NK cells, natural killer cells; cDCs, conventional dendritic cells.

**Additional file 6: Supplementary Table 1.** Demographic, clinical, molecular, and diagnostic information of all patients in this study.

**Additional file 7: Supplementary Table 2.** The known marker genes used for annotating cell clusters.

**Additional file 8: Supplementary Table 3.** Specific marker genes of control donor BM cells. BM, bone marrow.

**Additional file 9: Supplementary Table 4.** Specific marker genes of malignant plasma cell subclones in NDMM and RRMM patients. NDMM, newly diagnosed multiple myeloma; RRMM, relapsed and/or refractory MM.

**Additional file 10: Supplementary Table 5.** Specific marker genes of BM tumor microenvironment cells in patients with NDMM and RRMM.

**Additional file 11: Supplementary Table 6.** Characterization of specific marker genes for type I and type IX malignant progenitors.

**Additional file 12: Supplementary Table 7.** Relative abundance estimation and typing of type I and IX malignant progenitors in a large clinical cohort.

## Data Availability

All data generated or analysed during this study are included in this published article [and its supplementary information files].

## Abbreviations

MM: Multiple myeloma
BM: Bone marrow
scRNA-seq: Single-cell RNA sequencing
NDMM: Newly Diagnosed MM
RRMM: Relapsed and/or refractory MM
CD20^+^ RRMM: CD20-positive RRMM
PBS: Phosphate-buffered saline
UMIs: Unique molecular identifiers
t-SNE: t-distributed stochastic neighbor embedding
TF: Transcription factor
mDNAsi: DNA methylation-based stemness index
mRNAsi: Gene expression-based stemness index
SCENIC: Single cell regulatory network inference and clustering
RAS: Regulon activity score
RSS: Regulon-specific score
JS: Jensen–Shannon divergence
CSI: Connection specificity index
DEGs: Differentially expressed genes
CNV: Copy number variation
SNV: Single-nucleotide variant
TME: Tumor microenvironment
TMB: Tumor mutational burden
logFC: Log fold change
OS: Overall survival
RFS: Relapse-free survival
ROC: Receiver operator characteristic
SOM: Self-organizing mapping
PCs: Principal components
HSCs: Hematopoietic stem cells
DCs: Dendritic cells
DEL: Deletion
INS: Insertion
cDCs: Classical dendritic cells
HHV-8: Herpesvirus type 8
