# Supplementary figures and images for: Malignant clonal evolution drives multiple myeloma cellular ecological diversity and microenvironment reprogramming
Source: Mol Cancer. 2022 Sep 22;21:182. doi: 10.1186/s12943-022-01648-z (PMC9492468; doi:10.1186/s12943-022-01648-z)

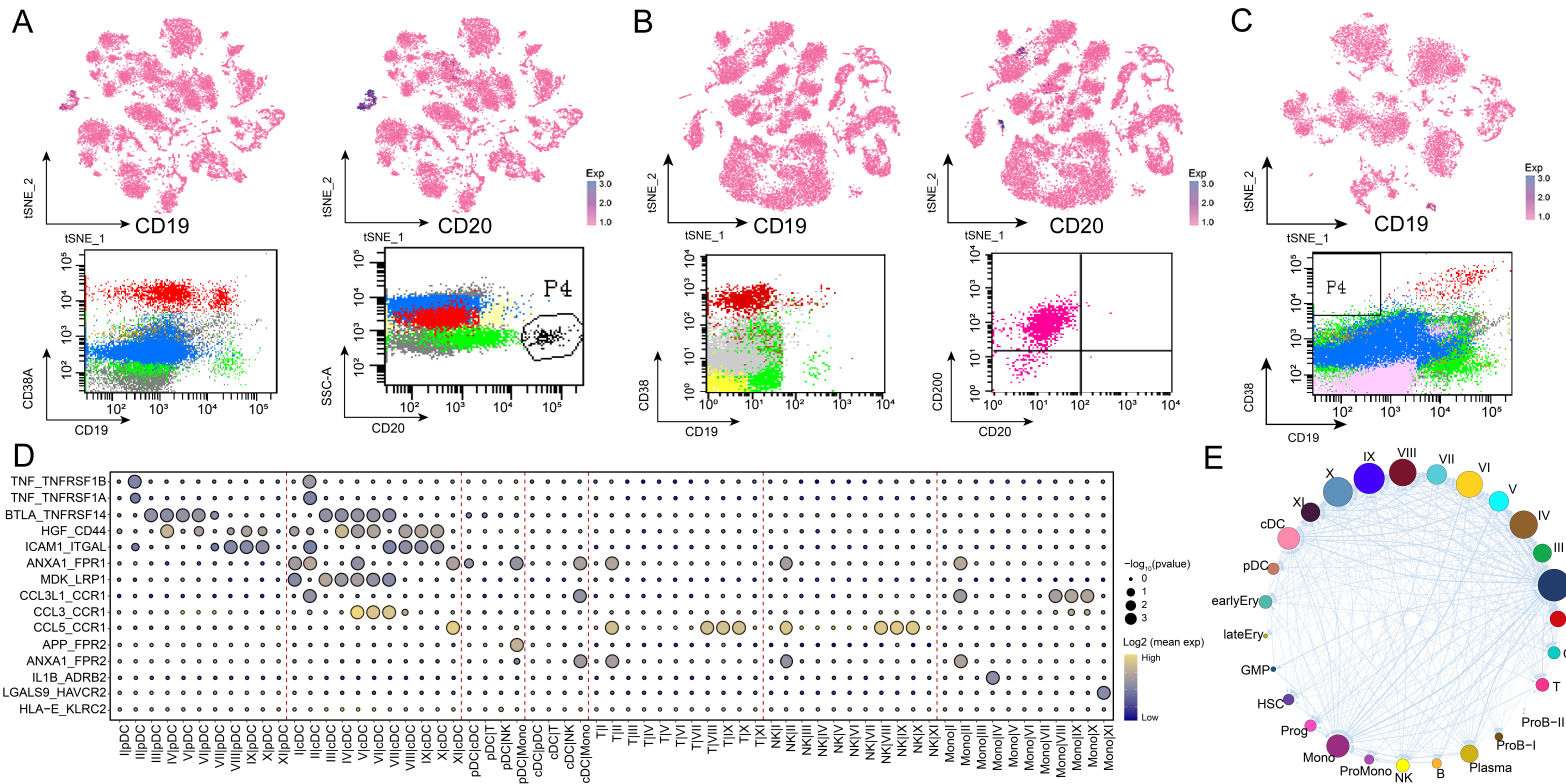

Supplement: Supplementary file 1 — Additional file 1: Supplementary Fig. 1. BM intercellular communication events in patients with NDMM and RRMM. (A and B) Expression patterns of CD19 and CD20 in single cells from NDMM (A) and RRMM (B) patients. Top: t-SNE single-cell atlas showing the expression of CD19 and CD20 in BM single cells from MM patients. Bottom: Verification of the expression of CD19 and CD20 in BM cells by flow cytometry. (C) Expression pattern of CD19 in single cells from CD20+ RRMM patients. Top: t-SNE single-cell atlas showing the expression of CD19 in BM single cells from CD20+ RRMM patients. Bottom: Verification of CD19 expression in BM cells from CD20+ RRMM patients by flow cytometry. (D) High-confidence receptor-ligand interaction for BM intercellular communication events in patients with NDMM and RRMM. Bubbles represent the average expression of high-confidence ligand-receptor interaction pairs in the source and target cells. (E) Overview of high-confidence intercellular communication networks in BM patients with NDMM and RRMM. Each bubble represents a cellular identity of the BM in NDMM and RRMM patients, and the coloring is consistent with that of the corresponding single-cell atlas. Bubble size represents active cell communication with other cells. Each arrow represents the interaction between the source cell ligand and the target cell receptor, and its thickness represents the number of ligand-receptor interaction pairs. All communication events were detected using any two tools among CellPhoneDB, iTalk, and CellCrosstalk, which represent the confidence of the reciprocal pair. BM, bone marrow; NDMM, newly diagnosed multiple myeloma; RRMM, relapsed and/or refractory MM; t-SNE, t-distribution and stochastic neighbor embedding. [file 12943_2022_1648_MOESM1_ESM.pdf]

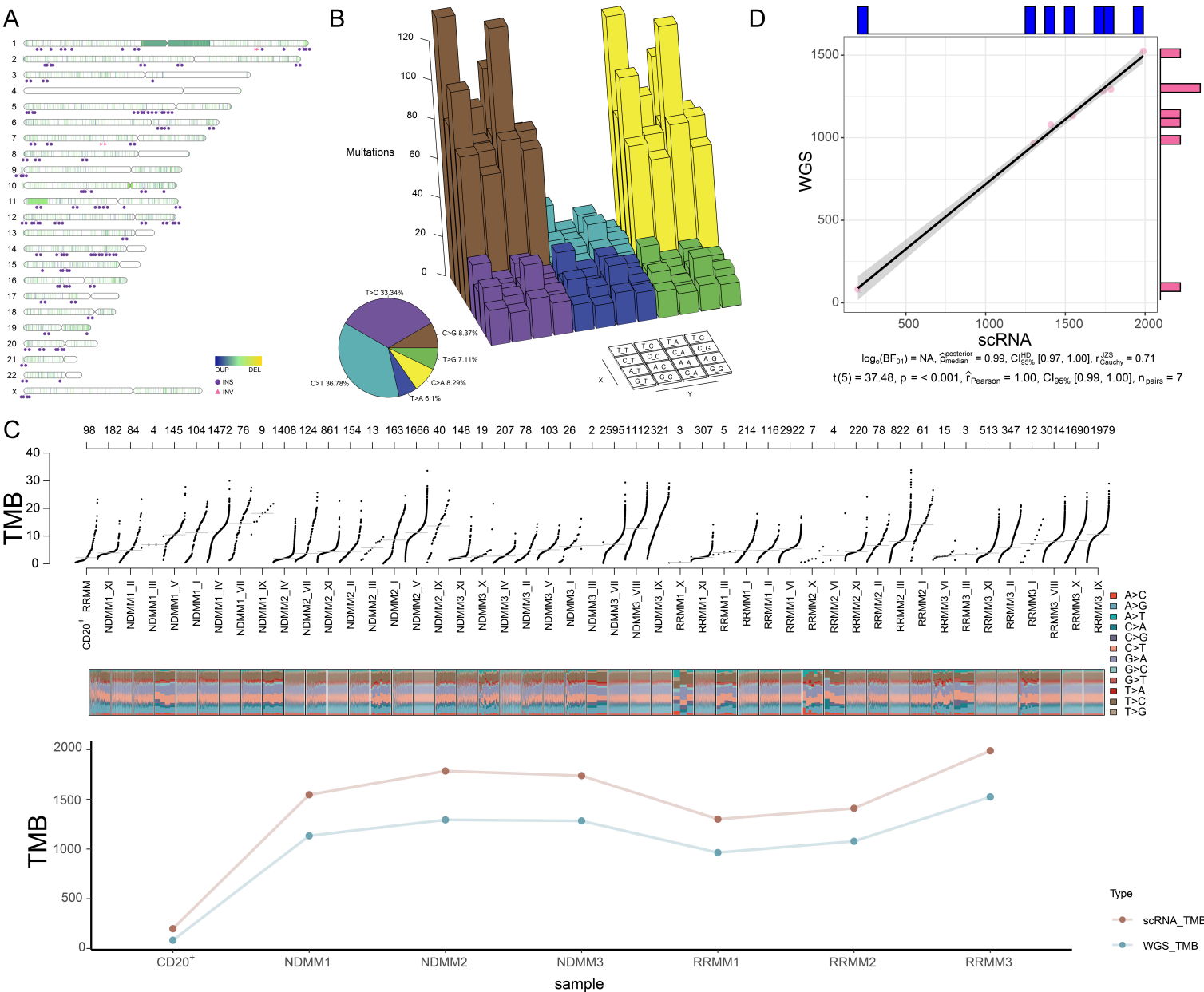

Supplement: Supplementary file 2 — Additional file 2: Supplementary Fig. 2. Nanopore sequencing combined with single-cell transcriptome to identify malignant driving events. (A) Diagram of genomic SVs in patients with NDMM and RRMM. “INS” and “INV” of patients with counts > 5 are displayed, and colors on chromosomes represent the number of patients with DEL (green to yellow) and DUP (green to blue). (B) Patterns of gene mutations in MM patients. (C) Volume and single-cell levels of the TMB in the BM of MM patients. Top: TMB levels of different malignant subclones. Middle: Gene mutation patterns in different malignant subclones. Bottom: TMB horizontal curve of patients with MM. (D) Correlation of TMB in MM patients at the bulk and single-cell levels. The BM TMB of MM patients was significantly correlated at the bulk level and at the single-cell level (P < 0.001), demonstrating the feasibility and reliability of detecting the SNV spectrum and TMB level at the single-cell level. SV, structural variation; INS, insert; INV, inversion; DEL, deletion; DUP, duplication; CNV, copy number variation; SNV, single nucleotide variation; TMB, tumor mutational burden. [file 12943_2022_1648_MOESM2_ESM.pdf]

A

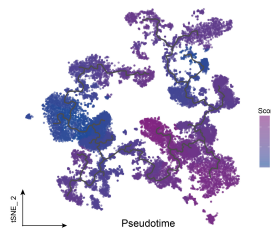

B

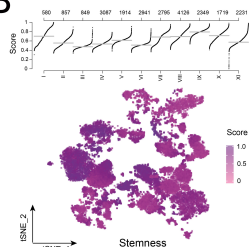

C

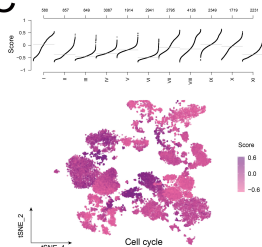

D

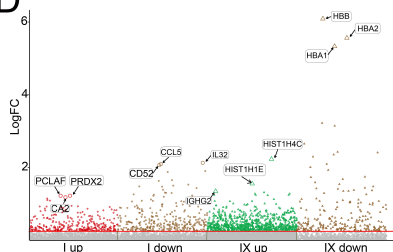

E

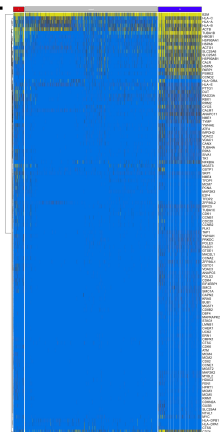

F

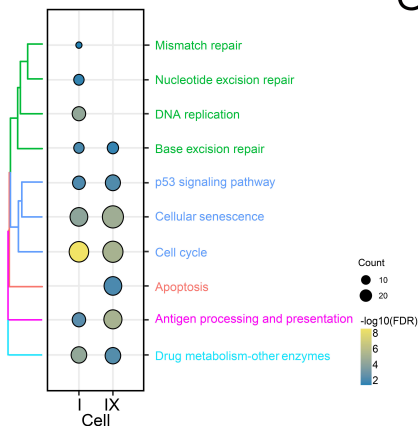

G

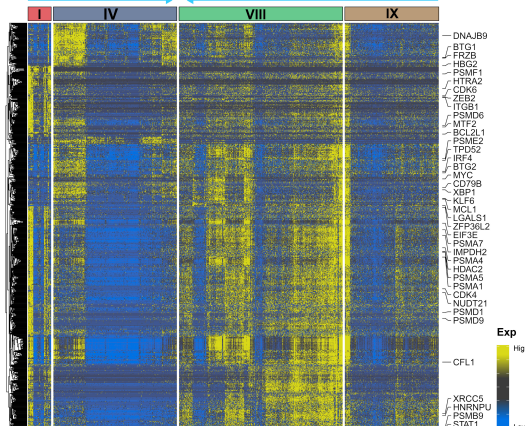

Supplement: Supplementary file 3 — Additional file 3: Supplementary Fig. 3. Exploring malignant origins from the perspective of clonal evolution. (A) Pseudotime trajectory of myeloid malignant subclones in NDMM and RRMM patients mapped in the t-SNE–based single-cell atlas. (B, C) Stemness score (B) and cell cycle score (C) of myeloid malignant subclones in patients with NDMM and RRMM. Top: Comparison of stemness and cell cycle scores between myeloid malignant subclones from patients with NDMM and RRMM. Bottom: Stemness and cell cycle scores of myeloid malignant subclones in NDMM and RRMM patients mapped using the t-SNE single-cell atlas. (D) Dysregulated expression patterns of malignant leader genes in type I and IX malignant origins compared to normal B cells. (E) Type I and IX malignant origin expression dysregulated genes are significantly involved in malignant precursor biological signals. These biological signals of malignant precursors can be grouped into five modules according to shared gene members. Each module was assigned an independent color. (F) Gene expression patterns of the evolution of malignant origins toward dominant subclones during natural development. (G) Expression patterns of dysregulated genes across Type I, IV, VIII and IX. [file 12943_2022_1648_MOESM3_ESM.pdf]

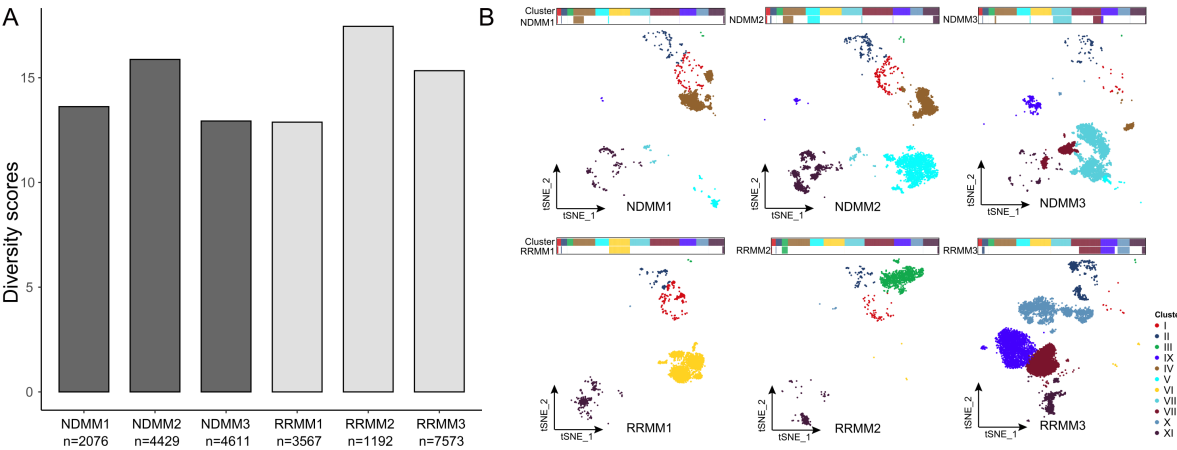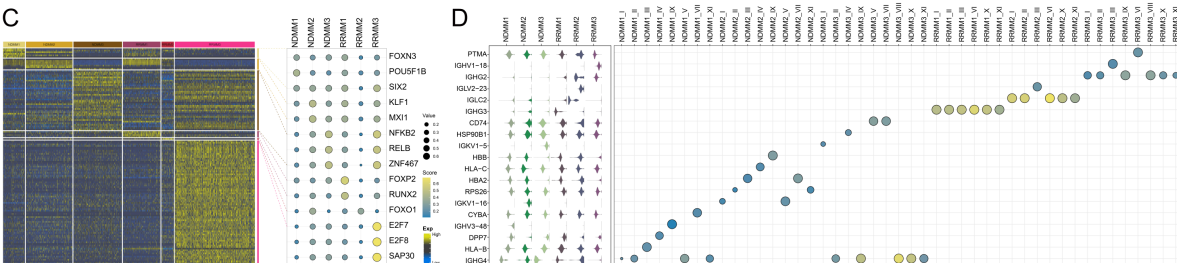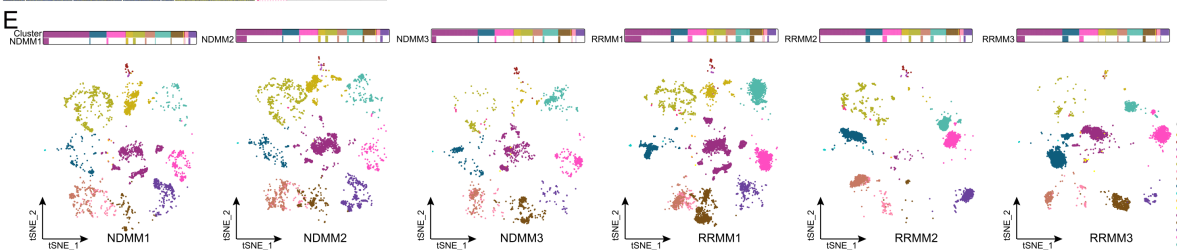

Supplement: Supplementary file 4 — Additional file 4: Supplementary Fig. 4. Cellular heterogeneity in the myeloma of MM patients. (A) Heterogeneity score of the myeloma in NDMM and RRMM patients. (B) Single-cell atlas based on t-SNE showing heterogeneity of malignant subclonal ecological components in different MM patients. (C) Specific markers of malignant subclones in patients with NDMM and RRMM. Left: Expression pattern of malignant subclonal-specific marker genes in patients. Right: TFs that regulate malignant subclonal-specific markers. (D) Contribution of cell subsets with malignant subclone-specific markers in patients. Left: Expression patterns of malignant subclonal-specific marker genes in patients. The malignant subclonal marker genes illustrated are those that are the most significant and associated with subsequent MM malignant plasma cell clonal evolution reprogramming of immune cells in the microenvironment. Right: Contribution of cell subsets specifically labeled by the top specific marker malignant subclones in patients. (E) The single-cell atlas based on t-SNE showing the heterogeneity of cell ecological components in the myeloma microenvironment in different MM patients. TF, transcription factor. [file 12943_2022_1648_MOESM4_ESM.pdf]

**A**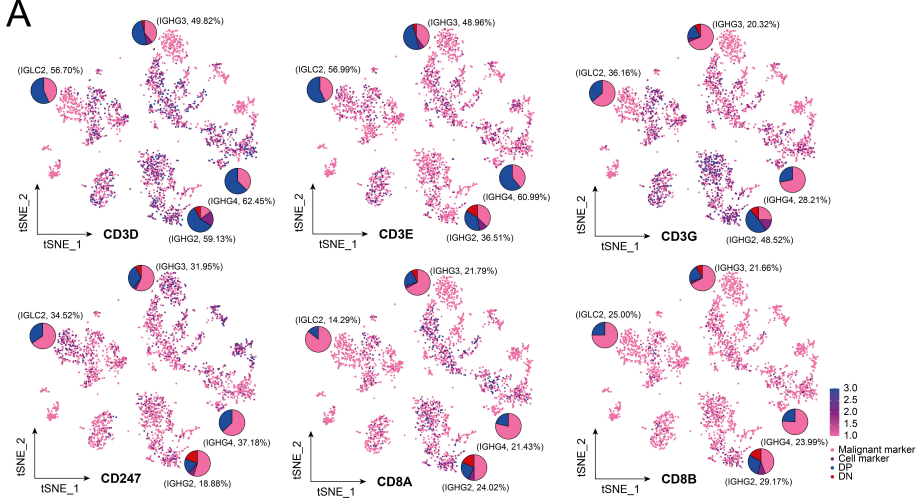**B**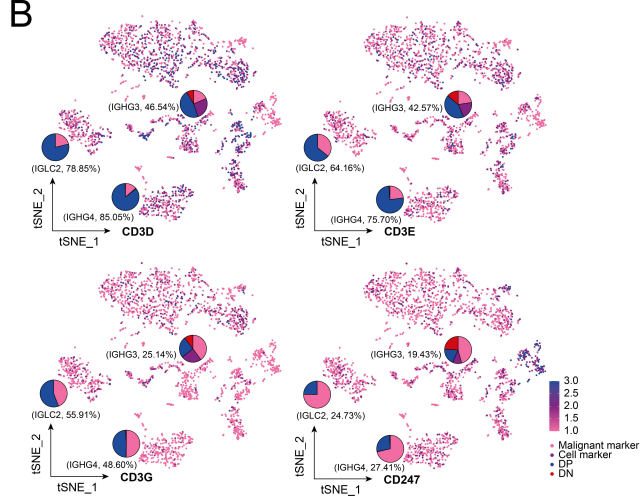**C**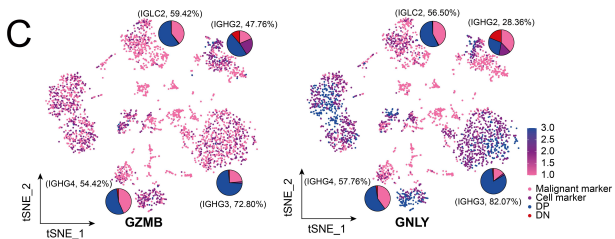**D**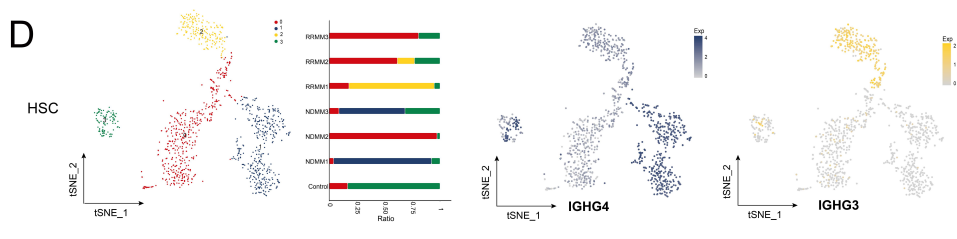**E**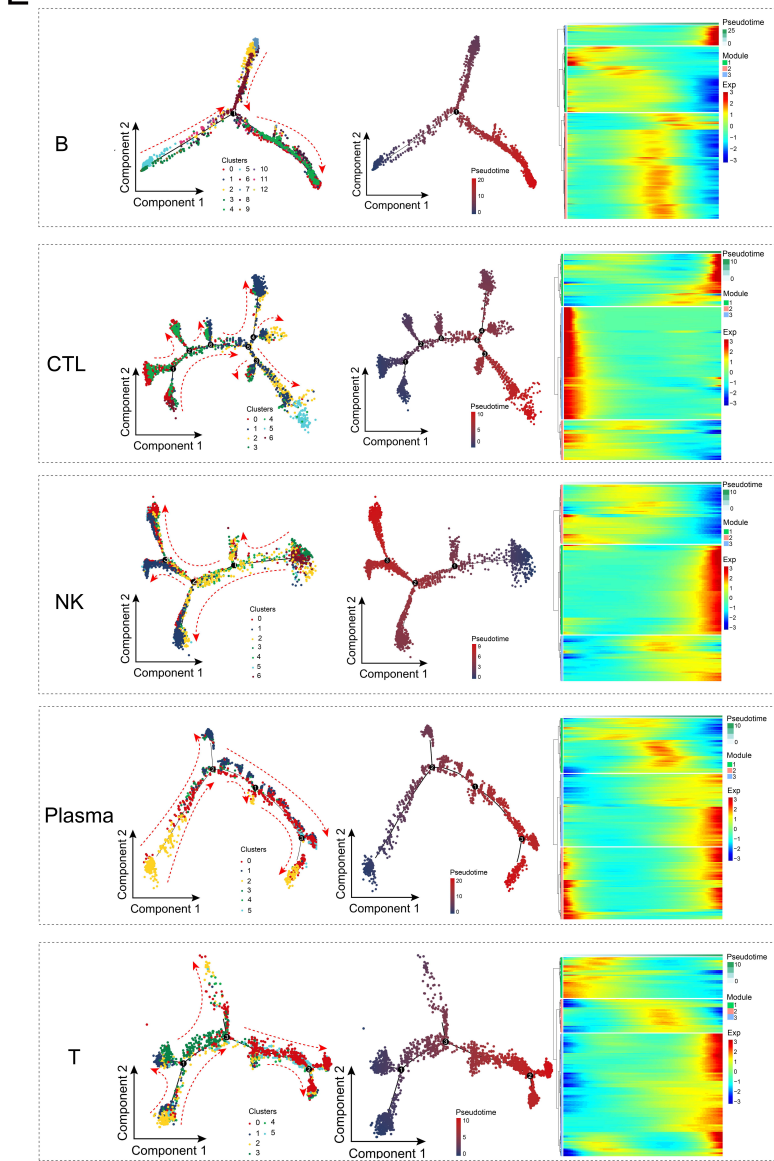**F**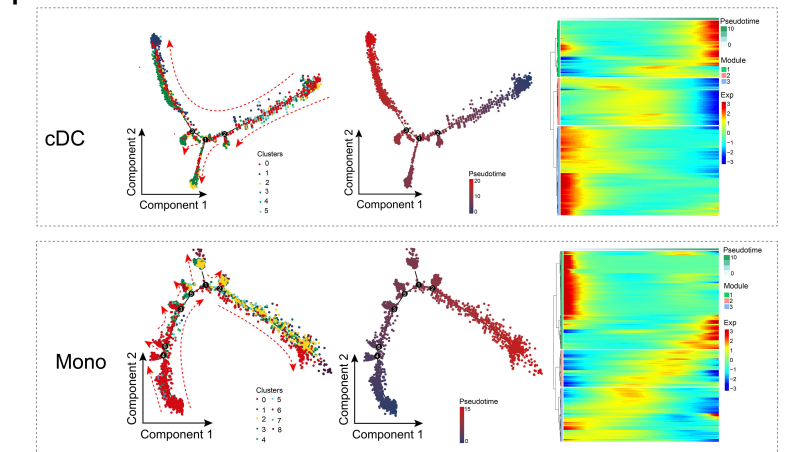**G**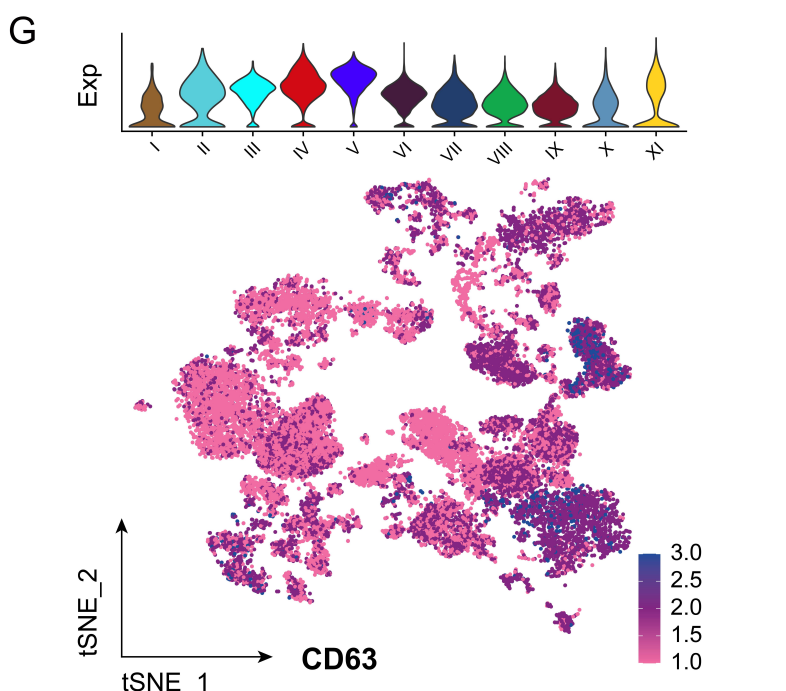

Supplement: Supplementary file 5 — Additional file 5: Supplementary Fig. 5. Expression patterns of immune cell subpopulations in MM. (A-C) Expression patterns of known cell identity-specific markers and their co-location with malignant markers in lymphocytes provide evidence for MM reprogramming of microenvironment cells. The pie chart shows the co-location between known cell identity-specific markers and corresponding patient heterogeneous malignant marker immunoglobulins. (D) HSC subset profiles of control donor BM and the MM patient BM microenvironment. Left: Single-cell subpopulation atlas of HSCs. Middle: Proportion of HSCs in control donors and different MM patients. Right: Expression patterns of cell subset-specific markers mapped on the HSC single-cell atlas. (E, F) MM malignant clonal evolution reprograms pseudotime trajectories and expression pattern changes in lymphoid (E) and myeloid cells (F). Lymphocytes include B cells, plasma, CTLs, T cells, and NK cells, whereas myeloid cells include cDCs and monocytes. For each cell type, the pseudotime trajectory (left), pseudotime value change (middle), and expression pattern change (right) from normal cells to MM during MM malignant clonal evolution reprogramming are shown for lymphocyte and myeloid cell subsets. (G) Expression patterns of the exosome-specific marker gene CD63 in malignant cells. HSCs, hematopoietic stem cells; CTLs, cytotoxic T lymphocytes; NK cells, natural killer cells; cDCs, conventional dendritic cells. [file 12943_2022_1648_MOESM5_ESM.pdf]
